# Fenofibrate reduces cisplatin-induced apoptosis by inhibiting the p53/Puma/Caspase-9 pathway and the MAPK/Caspase-8 pathway rather than by promoting autophagy in murine renal proximal tubular cells

**DOI:** 10.1016/j.bbrep.2022.101237

**Published:** 2022-02-28

**Authors:** Hideki Kimura, Kazuko Kamiyama, Toru Imamoto, Izumi Takeda, Shinya Masunaga, Mamiko Kobayashi, Daisuke Mikami, Naoki Takahashi, Kenji Kasuno, Takeshi Sugaya, Masayuki Iwano

**Affiliations:** a Department of Clinical Laboratory, University of Fukui Hospital, Fukui, Japan; b Division of Nephrology, Department of General Medicine, School of Medicine, Faculty of Medical Sciences, University of Fukui, Fukui, Japan; c Cimic Corporation, Tokyo, Japan

**Keywords:** Renal tubular cells, Cisplatin, Fenofibrate, Apoptosis, Mitochondrial and death receptor pathways, Autophagy

## Abstract

The main lesion of cisplatin nephrotoxicity is damage to proximal tubular cells due to increased apoptosis via the mitochondrial and death receptor pathways, which may be alleviated by appropriate promotion of autophagy. Fenofibrate, a peroxisome proliferator-activated receptor-alpha (PPAR-α) activator, is recently reported to promote autophagy as well as protect against cisplatin nephrotoxicity, although the mechanisms were only partially analyzed. Here, the detailed mechanisms of these putative protective effects were investigated in a murine renal proximal tubular (mProx) cell line. Fenofibrate attenuated cisplatin-induced apoptosis of mProx cells based on flow cytometry. As for the mitochondrial apoptotic pathway, the reagent reduced cisplatin-stimulated caspase-3 activation by decreasing the phosphorylation of p53, JNK, and 14-3-3, cytosolic and mitochondrial Puma accumulation, cytochrome C release to the cytosol, and resulting cytosolic caspase-9 activation. Fenofibrate also decreased cisplatin-stimulated activation of caspases-8 by suppressing MAPK and NFkB pathways and reducing the gene expression of TNF-α, TL1A, and Fas, main mediators of the death receptor apoptotic pathway. Autophagy defined by p62 reduction and an increase in LC3 II/I was promoted by fenofibrate in mProx cells under starvation. Autophagy inhibition using 3-MA further increased basal and cisplatin-induced caspase-3 and -8 activation, but had no influence on the inhibitory effects of fenofibrate on caspase activation. In conclusion, our study suggests fenofibrate to be a candidate agent to mitigate cisplatin nephrotoxicity by inhibiting the mitochondrial and death apoptotic pathways rather than by promoting autophagy.

## Highlights

• Fenofibrate reduced cisplatin-induced apoptosis in mProx cells.
• Fenofibrate reduced caspase-3 activation by inhibiting p53/Puma/caspase-9 pathways.
• Fenofibrate reduced caspase-8 activation by inhibiting MAPK/death receptor pathways.
• Fenofibrate promoted autophagy in mProx cells under starvation conditions.
• Autophagy inhibition, however, did not affect the protective effects.

## 1 Introduction

Cisplatin is widely and commonly used as a chemotherapeutic agent for the treatment of solid tumors, but the frequent occurrence of renal injury is the major limitation of cisplatin-based chemotherapy [1,2]. Renal proximal tubular damage due to activation of cell death and inflammatory pathways pathophysiologically characterizes the cisplatin nephrotoxicity [1,2]. The tubular damage develops due to apoptosis through the intrinsic mitochondrial and endoplasmic reticulum (ER) pathway and the extrinsic pathway activated by death receptors via inflammatory cytokines such as TNF-α [2]. As for the intrinsic pathway, cisplatin induces p53-stimulated mitochondrial damage followed by the activation of caspase-9 and then caspase-3, and also induces ER damage followed by caspase-12 activation, finally producing apoptosis in cisplatin nephrotoxicity [[3], [4], [5]]. The death receptor pathways highly involved in caspase-8 activation is stimulated by cisplatin-enhanced activation of mitogen-activated protein kinases (MAPK), including c-JUN N-terminal kinase (JNK), extracellular signal-regulated kinase (ERK), and p38 kinase (p38) [1,2]. Furthermore, cisplatin promotes autophagy before apoptosis in cultured renal tubular cells [6] and in mouse kidneys [7]. Notably, promotion of autophagy protects against cisplatin-induced tubular cell injury [8], whereas inhibition of autophagy worsens cisplatin nephrotoxicity both *in vitro* [6] and *in vivo* [7,9].

Fenofibrate is a fibric acid derivative (fibrate) that has been used worldwide as an anti-hyperlipidemic agent to improve dyslipidemia through the activation of peroxisome proliferator-activated receptors-α (PPAR-α) as a ligand [10]. Cell-protective effects of several fibrates were reported to be due to pleiotropic effects. WY-14,643, a fibrate, and fenofibrate reduce cisplatin-induced nephrotoxicity through down-regulating pro-inflammatory cytokines and inhibiting MAPK activation that are dependent on and independent of PPAR-α activation, respectively [11,12]. Another fibrate, bezafibrate, prevents cisplatin-induced proximal tubule cell apoptosis via suppressing mitochondrial Bax accumulation and consequent caspase-3 activation [13]. In renal tubular cells, fenofibrate induces autophagy in a PPAR-α-independent manner [14], although PPAR-α activation promotes autophagy in the mouse liver [15]. Most recently, we reported that GW0742, an activator of PPAR-γ which promotes mitochondrial biogenesis, reduced cisplatin-induced injury in marine renal tubular cells but had no autophagy-enhancing effect [16].

Considering these previous studies, fenofibrate may guard against cisplatin nephrotoxicity through the regulation of inflammation and autophagy regardless of PPAR-α activation. However, the detailed mechanisms of the protective effects remain unknown, especially regarding the involvement of mitochondrial and death receptor pathways and autophagy. Speaking more exactly, how fenofibrate regulates p53/caspase-9/caspase-3 pathway and MAPK/the death receptor/caspase-8 pathway in cisplatin nephrotoxicity has not yet been clarified at all. In this study, therefore, we investigated the protective effects of fenofibrate and their molecular mechanisms, focusing these unknowns, on cisplatin-induced injury in a murine renal proximal tubular (mProx) cell line.

## 2 Materials and methods

### 2.1 Materials

Fenofibrate and cisplatin (SIGMA-Aldrich, St. Louis, MO, USA) were used. Rabbit monoclonal antibodies against human p53 upregulated modulator of apoptosis (Puma), mouse cleaved caspase-8, human p38 and human NFkB, phosphorylated human JNK (p-JNK), human p38 (p-p38), human ERK (p-ERK), human NFkB (p-NFkB) and human 14-3-3 (p-14-3-3), and rabbit polyclonal antibodies against human cleaved caspase-3, human JNK, rat ERK, human 14-3-3, mouse cleaved caspase-9, mouse caspase 12, human AMPK and human LC3 were purchased from Cell Signaling Technology (Boston, USA). Rabbit polyclonal antibody against human p62 was purchased from MEDICAL and BIOLOGICAL LABORATORIES Co. Ltd. (Nagano, Japan). Rabbit polyclonal antibodies against β-actin, and rabbit monoclonal antibodies against human Cox IV were purchased from Abcam Inc. (Cambridge, UK). Horseradish peroxidase (HRP)–conjugated anti-mouse or anti-rabbit immunoglobulins (Dako, Glostrup, Denmark) were also used. An inhibitor of autophagy, 3-methyladenine (3-MA), was purchased from Santa Cruz Biotechnology (Texas, U.S.A).

### 2.2 Tubular cell cultures

mProx cells were generated as previously described [17]. These cells were grown in modified K-1 medium (50:50 Ham's F-12/DMEM) with 10% FBS, 5% CO2 and 95% air in a humidified atmosphere at 37.0 °C. mProx cells (passage 10 through 14) were seeded on 12-well plates. The modified K-1 medium was renewed every 2 days until semi-confluence was achieved. For starvation experiments, mProx cells were incubated in HBSS medium with Ca2+ and Mg2+ (Wako Pure Chemical Industries, Ltd) for 3 or 6 h as previously reported [15,18]. Cisplatin was added to the medium at a final concentration of 5, 10 or 25 μM for 24 h mProx cells were also treated with cisplatin (25 μM) for 0, 3, 6, 16 or 24 h. To investigate the effects of fenofibrate and its dose-dependency, mProx cells were treated for 24 h with simultaneous administration of cisplatin (25 μM) and fenofibrate (0, 10, 25 or 50 μM). To investigate the effects of autophagy inhibition on cisplatin-induced apoptosis, mProx cells were treated with cisplatin (25 μM) for 24 h in the presence or absence of fenofibrate (50 μM) or an inhibitor of a late phase of autophagy, 3-MA (3 mM). Fenofibrate, cisplatin and 3-MA were prepared as stock solutions in dimethyl sulfoxide (DMSO), 0.9% NaCl and modified K1 medium, respectively, and then further diluted. The final concentration of DMSO did not exceed 0.1%.

### 2.3 Annexin V-FITC/Propidium Iodide (PI) assay

To detect phosphatidylserine translocation from the inner to the outer plasma membrane as a marker of an early stage of apoptosis, the Annexin V-FITC kit (MBL CO. LTD., Nagoya, Japan) was used according to the manufacturer's protocol. Briefly, mProx cells grown on a 12-well plate were treated for 24 h with or without cisplatin (25 μM) in the presence or absence of fenofibrate (10, 25 and 50 μM). Harvested cells with trypsinization were washed with phosphate-buffered saline, and incubated in a binding buffer containing annexin V and PI for 15 min at room temperature in the dark. The stained cells were analyzed by flow cytometry using fluorescence-activated cell sorting (BD FACSCanto II, BD Biosciences, San Jose, CA, USA) and CellQuest software (BD Biosciences), with the acquisition of a total 10,000 events/sample to ensure sufficient data.

### 2.4 TaqMan real-time PCR assay

The TaqMan real-time PCR assay was performed as previously reported [19]. Unlabeled specific primers and TaqMan MGB probes (6-FAM dye-labeled) were purchased from Applied Biosystems to target mouse TNF-α (Assay ID: Mm00443258_m1). mRNA amounts of each gene were normalized to β2-microglobulin mRNA levels. The average mRNA amount of each gene in the untreated cells was set to 1.0.

### 2.5 Immunoblot analysis

Whole cell lysates were prepared using RIPA buffer containing phosphatase inhibitors (Santa Cruz Biotechnology, CA, USA), and mitochondrial and cytosolic fractions of mProx cells were prepared using EzSubcell Fraction (ATTO corp., Osaka, Japan) according to the manufacturer's instructions. Protein lysates (10 μg) were analyzed by immunoblot analysis as previously reported [19]. The membranes to which proteins were transferred were incubated with anti-cleaved caspases-3 (1:1000), anti-cleaved caspase-8 (1:1000), anti-cleaved caspase-9 (1:1000), anti-JNK (1:1000), ant-p-JNK (1:1000), anti-p38 (1:1000), anti-p-p38 (1:1000), anti-ERK (1:1000), anti-p-ERK (1:1000), anti-NFkB (1:1000), anti-p-NFkB (1:1000), anti-AMPK (1:1000), anti-p-AMPK (1:1000), anti-p62 (1:1000), anti-LC3 (1:500), anti-Puma (1:1000), anti-Cox IV (1:2000) and anti-β actin (1:6000) antibodies for 20 min at room temperature. The membranes were then incubated with appropriate horseradish peroxidase-conjugated anti-mouse or anti-rabbit immunoglobulins (1:1000) at room temperature for 1 h. The detection of secondary antibodies was performed using ECL reagents. Protein amounts in the cytosol and cell lysates, and those in the mitochondria were normalized to β-actin and COX IV, respectively. The average target protein amount in the untreated cells was expressed as arbitrary values.

### 2.6 Statistical analyses

All data are presented as the means ± standard deviation (±SD). The Student's t-test or 1-way analysis of variance (ANOVA) was used to evaluate the significance of differences between two groups or among more than 3 groups, respectively. A P-value of <0.05 was considered significant.

## 3 Results

### 3.1 Fenofibrate attenuated cisplatin-induced apoptosis by reducing caspase-3 and -8, but not -12, activation

**Fig. 1:**
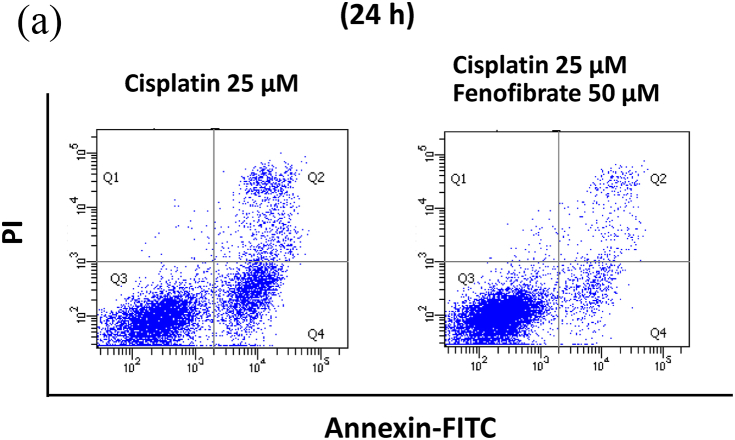

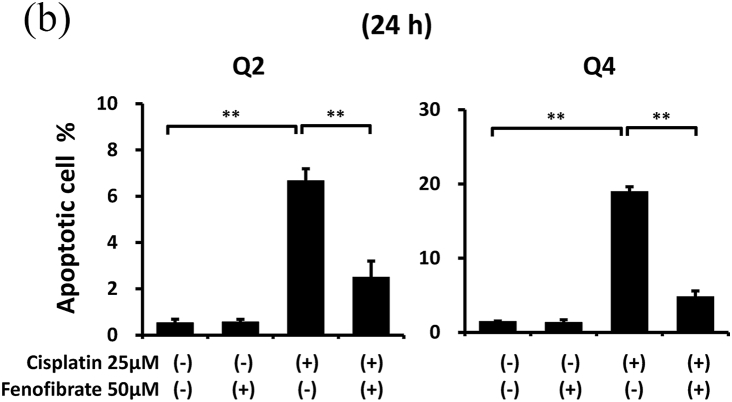

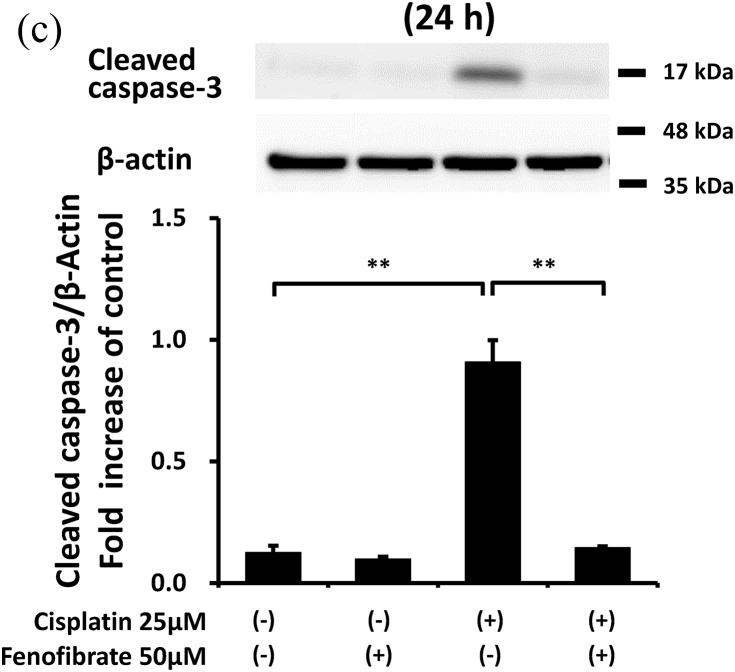

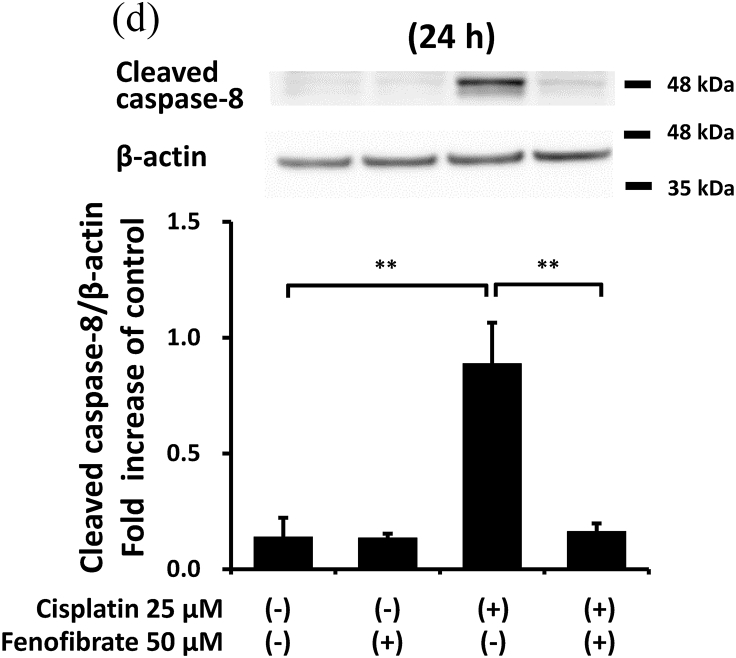
Fenofibrate attenuated cisplatin-induced apoptosis by reducing caspase-3 and -8 activation in mProx cells. **(A-D**) mProx cells were treated for 24 h with or without cisplatin (25 μM) in the presence or absence of fenofibrate (50 μM). (**A, B**) Harvested cells were stained with Annexin-FITC and PI, and analyzed by flow cytometry using fluorescence-activated cell sorting with the acquisition of a total 10,000 events/sample. (**B**) Percentages of Q2 and Q4 were calculated from data of each well sample. Results are expressed as the mean ± SD of a representative experiment (n = 4). Cleaved caspase-3 (**C**) and caspase-8 (**D**) amounts in cell lysates were measured by immunoblot analyses in the mProx cells incubated under the indicated conditions. A representative blot is shown in the upper panel (**C, D**). Results are expressed as the mean ± SD of a representative experiment (n = 3). **P < 0.01, significantly different from cells incubated under the indicated conditions, according to 1-way ANOVA with Scheffe's (**B-D**) post hoc comparisons.

When apoptotic cells were quantified by flow cytometry using Annexin V and PI staining, fenofibrate reduced cisplatin-induced apoptosis dose-dependently (data not shown for 10 μM and 25 μM, and Fig. 1A and B for 50 μM). Fenofibrate (50 μM) significantly reduced cisplatin-induced apoptosis from 6.7% to 2.5% in Q2 (a late phase of apoptosis) and from 19.0% to 4.8% in Q4 (an early phase of apoptosis) in the mProx cells (Fig. 1A and B). Cisplatin treatment for 24 h produced a dose-dependent increase in cleaved caspase-3 as a common apoptotic pathway, reaching a maximal value of over 10-fold at 25 μM (Fig. S1). Cisplatin (25 μM) time-dependently promoted caspase-3 activation at 16 and 24 h (Fig. S2). Fenofibrate significantly increased the inhibition rates of the caspase-3 and -8 activation dose-dependently in mProx cells treated with cisplatin (25 μM) for 24 h (Figs. S3A and S3B, 1C, and 1D), whereas it did not reduce caspase-12 activation (Fig. S4). Based on these results, cisplatin (25 μM) treatment for 24 h and simultaneous administration of fenofibrate (50 μM) were mainly used in the experiments performed below. Fenofibrate reduced cisplatin-induced apoptosis mainly by suppressing the activation of caspase-3 and-8 rather than caspase-12.

### 3.2 Fenofibrate reduced caspase-3 activation via the suppression of p53/Puma/cytochrome C pathways

In order to clarify how fenofibrate reduces cisplatin-induced activation of caspase-3, we first examined the effects on mitochondrial apoptotic pathways in mProx cells with or without cisplatin treatment in the presence or absence of fenofibrate.

**Fig. 2:**
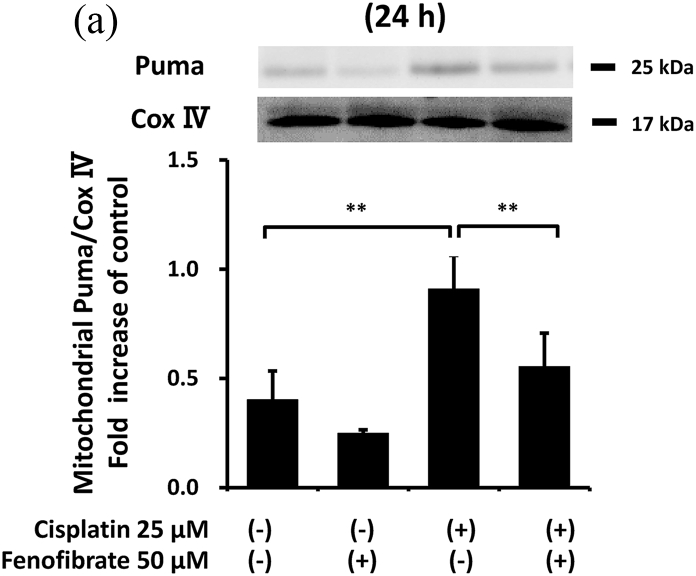

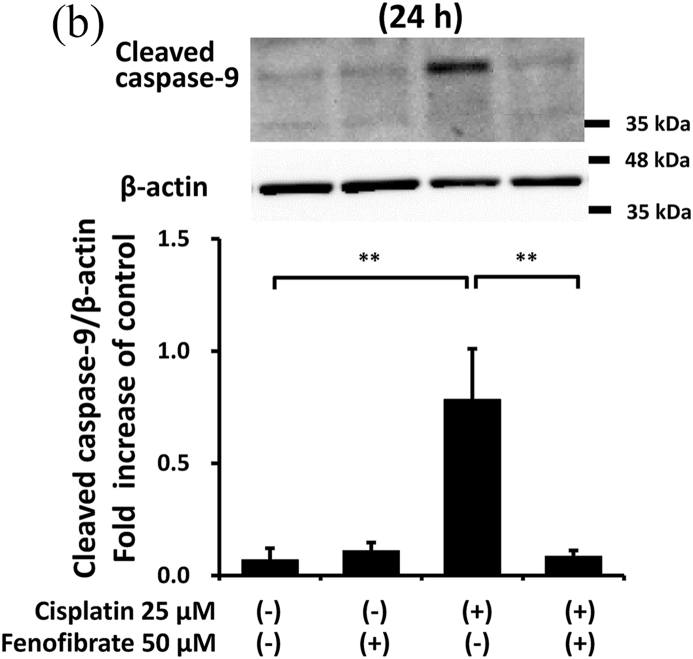

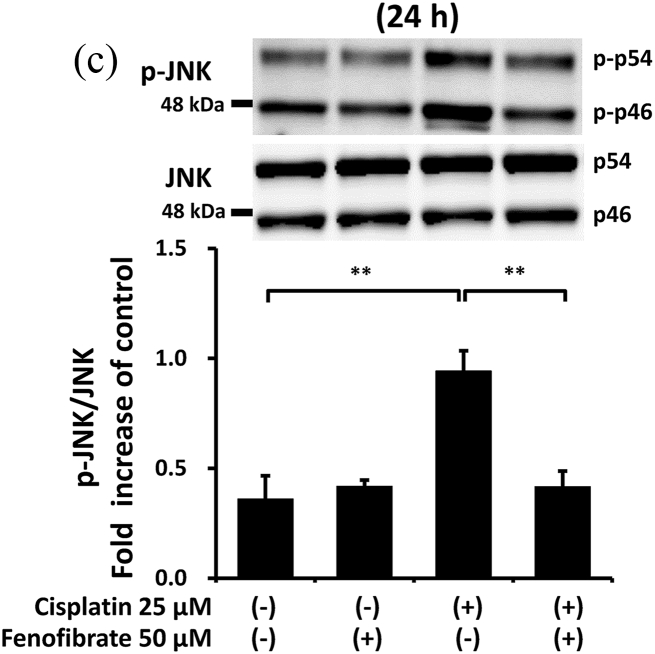

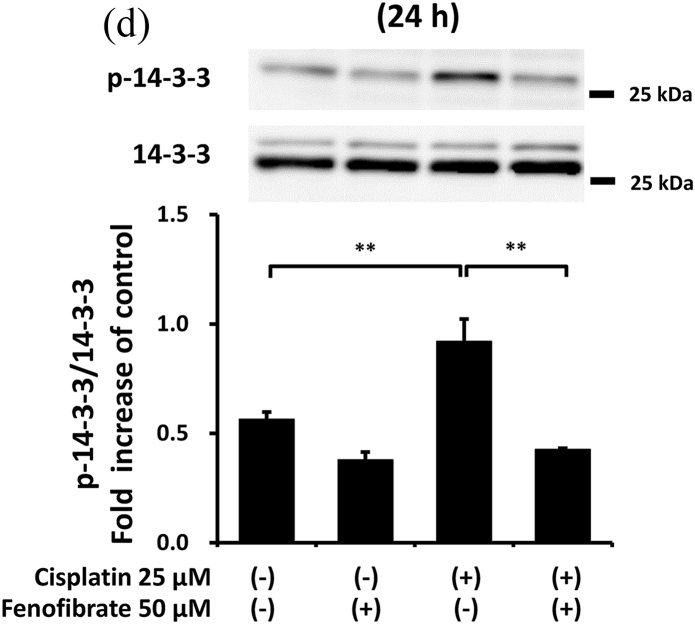
Fenofibrate reduced cisplatin-induced mitochondrial damage through the suppression of mitochondrial Puma/caspase-9 pathways in mProx cells. mProx cells were treated for 24 h with cisplatin (25 μM) in the presence or absence of fenofibrate (50 μM). Whole cell lysates and cytosolic and mitochondrial fractions were prepared using methods described in the Method's section. The amounts of mitochondrial Puma **(A)**, cleaved caspase-9 (**B**), JNK and phosphorylated JNK (p-JNK) (**C**), 14-3-3 and phosphorylated 14-3-3 (p-14-3-3) **(D)**, β-actin and Cox IV were measured by immunoblot analyses. Each protein amount was normalized to the indicated antigen levels. A representative blot is shown in the upper panel (**A-D**). Results are expressed as the mean ± SD of a representative experiment (n = 3 for each group). **P < 0.01, significantly different from cells incubated under the indicated conditions, according to 1-way ANOVA with Scheffe's (**A-D**) post hoc comparisons.

Cisplatin significantly increased the amount of phosphorylated p53 (p-p53) by 2.1-fold (Fig. S5), Bax mRNA by 1.7-fold (Fig. S6), Puma mRNA by 2.9-fold (Fig. S7), cellular Puma amount by 4.3-fold (Fig. S8) and mitochondrial Puma amount by 2.3-fold (Fig. 2A). Cisplatin also increased the cytosolic cytochrome C amount by 1.5-fold (Fig. S9) and cleaved caspase-9 amount by 9-fold (Fig. 2B). Fenofibrate significantly attenuated the cisplatin-induced production of p-p53 by 48% and Puma mRNA by 65% (Figs. S5 and S7). Fenofibrate also significantly attenuated the cisplatin-caused cellular and mitochondrial Puma amounts by 65% and 71%, respectively (Figs. S8 and 2A), and cytosolic cytochrome C release (Fig. S9) by 92%. It also significantly reduced the cleaved caspase-9 amount by 97% (Fig. 2B).

We next investigated other mediators of mitochondrial damage. As shown in Fig. 2C and D, fenofibrate reduced cisplatin-induced p-JNK and p-14-3-3, which may promote mitochondrial Bax and Bad translocation [20], although we were not able to detect the reduction of mitochondrial Bax translocation by fenofibrate (data not shown). Fenofibrate reduced the amounts of cisplatin-induced PDK4 mRNA and p-PDH antigen, which may cause mitochondrial dysfunction [21] (Figs. S10 and S11), and increased cisplatin-reduced mRNA levels of catalase, SOD2 and PPAR-α, which may reduce oxidative stress and lipid accumulation [21] (Figs. S12, S13 and S14).

Fenofibrate also reduced caspase-8 activation by inhibiting MAPK, NFkB and death receptor pathways.

To clarify the mechanisms by which fenofibrate attenuated cisplatin-induced activation of caspase-8, we next investigated the effects on inflammation and death receptor pathways in mProx cells treated with cisplatin.

**Fig. 3:**
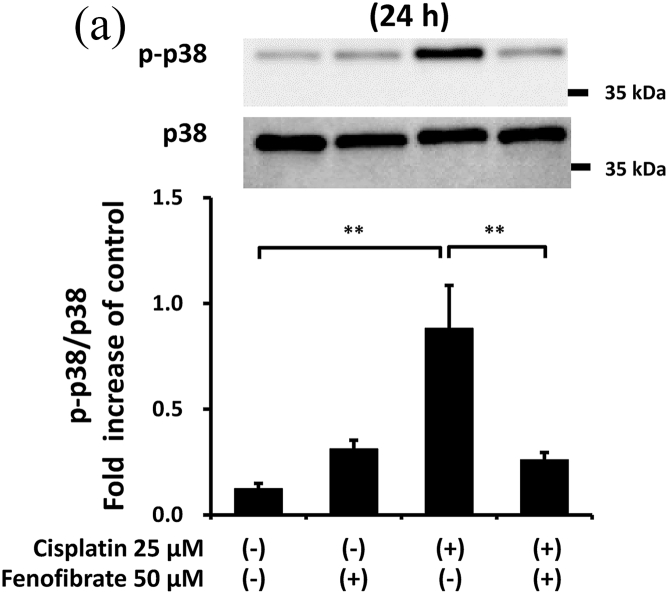

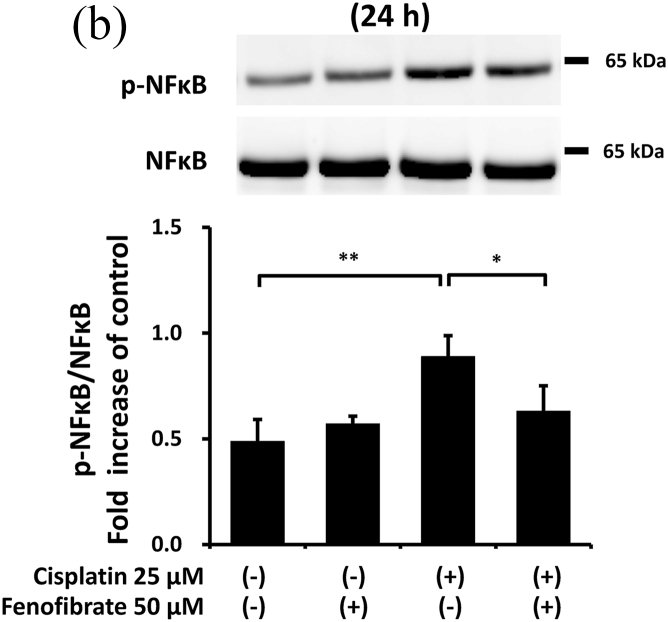

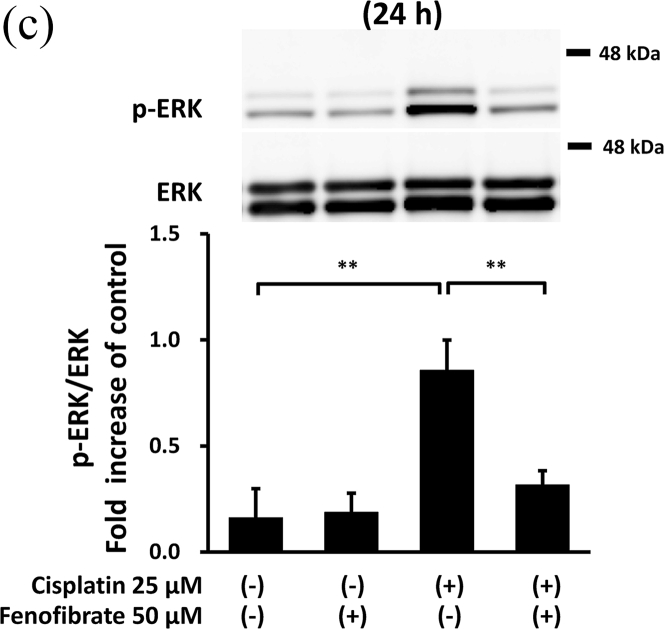

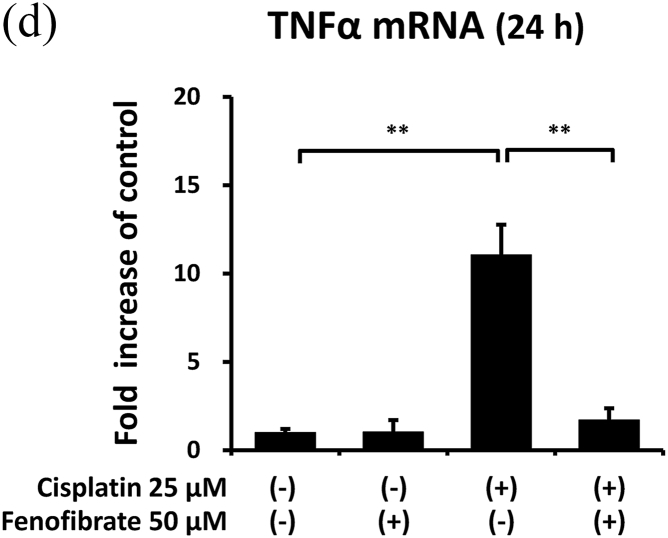
Fenofibrate reduced cisplatin-induced caspase-8 activation through the suppression of MAPK, NFkB and death receptor pathways in mProx cells. mProx cells were treated for 24 h with cisplatin (25 μM) in the presence or absence of fenofibrate (50 μM). Whole cell lysates were prepared for immunoblot analysis. The amounts of p38 and phosphorylated p38 (p-p38) (**A**), NFkB and phosphorylated NFkB (p-NFkB) **(B),** and ERK and phosphorylated ERK (p-ERK) **(C)** were measured by immunoblot analyses. Each phosphorylated protein amount was normalized to the levels of the corresponding protein. A representative blot is shown in the upper panel (**A-C**). The levels of TNF-α mRNA **(D)** were measured by real-time PCR assay and normalized to the levels of β2-microglobulin mRNA. The average level of each mRNA in untreated cells was set to 1.0. Results are expressed as the mean ± SD of a representative experiment (n = 3 for each group). *P < 0.05, **P < 0.01, significantly different from cells incubated under the indicated conditions, according to 1-way ANOVA with Scheffe's (**A, C, D**) or Fisher's (**B**) post hoc comparisons.

Cisplatin significantly increased the amounts of p-p38 by 7.3-fold (Fig. 3A), p-NFkB by 1.8-fold (Fig. 3B), p-ERK by 5.3-fold (Fig. 3C), TNF-α mRNA by 11.0-fold (Fig. 3D), TL1A mRNA by 2.4-fold (Fig. S15) and FAS mRNA by 11.8-fold (Fig. S16). In the cisplatin-treated mProx cells, fenofibrate reduced the amount of p-p38 by 81% (Fig. 3A), p-NFkB by 65% (Fig. 3B), p-ERK by 77% (Fig. 3C), TNF-α mRNA by 93% (Fig. 3D), TL1A mRNA by almost 100% (Fig. S15) and Fas mRNA by 95% (Fig. S16). In addition, c-FLIP, an inhibitor of caspase-8 activation through self-degradation [22], was analyzed. Fenofibrate also increased the cisplatin-reduced c-FLIP protein amount by 1.6-fold (Fig. S17), suggesting that the reduced c-FLIP degradation reflected fenofibrate-reduced caspase-8 activation.

Thus, fenofibrate reduced cisplatin-induced caspase-8 activation mainly by suppressing the activation of MAPK, NFkB and death receptor pathways.

### 3.3 Fenofibrate promoted autophagy in mProx cells

**Fig. 4:**
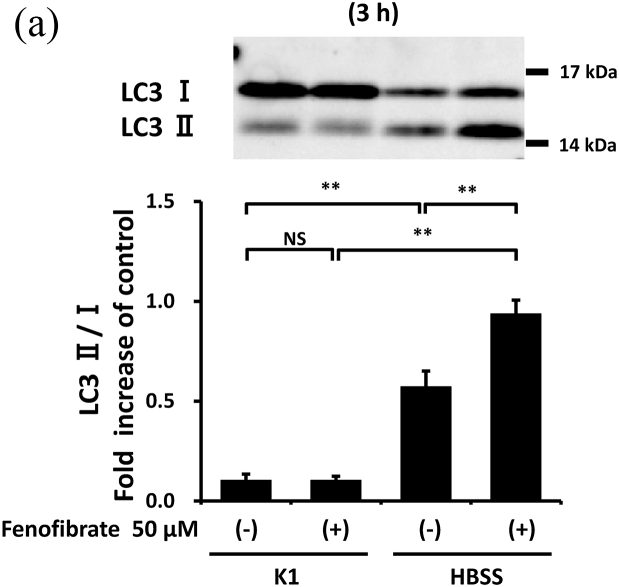

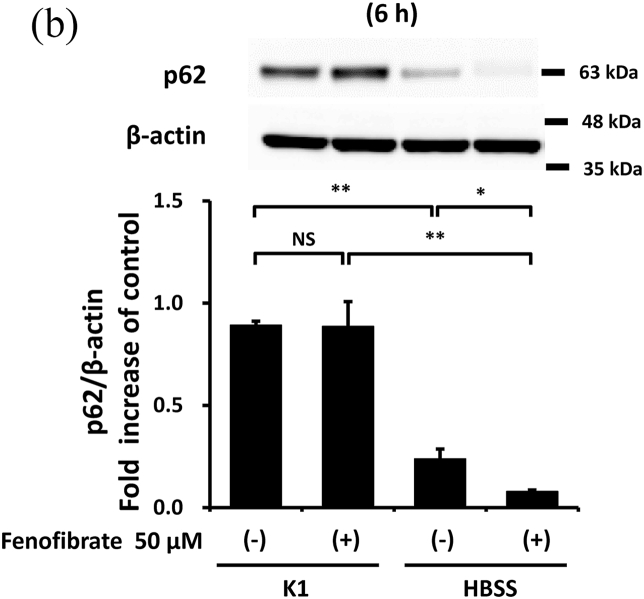

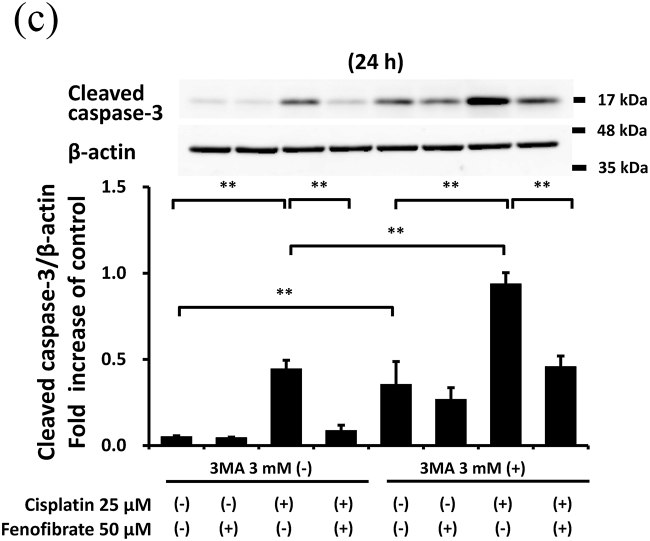

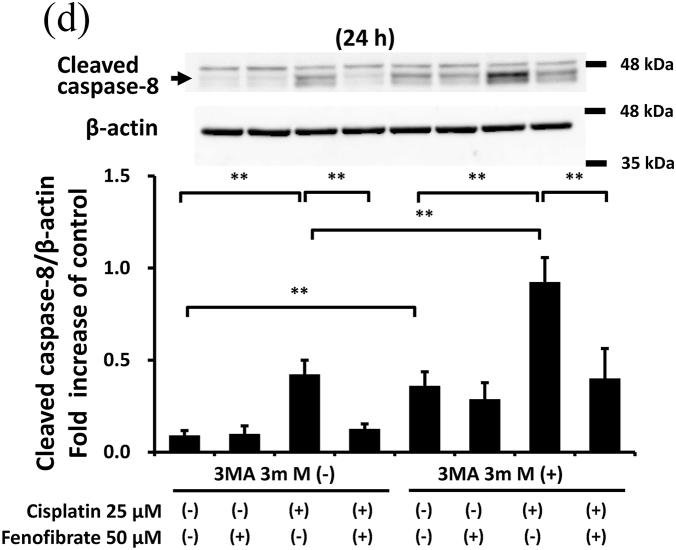
Fenofibrate reduced cisplatin-induced caspase-3 and -8 activation with little involvement of autophagy promotion despite its autophagy-promoting effects. (**A, B**) Confluent mProx cells were incubated for 3 or 6 h in modified K-1 medium or HBSS medium in the presence or absence of fenofibrate (50 μM). (**C, D**) mProx cells were treated for 24 h with cisplatin (0 or 25 μM) in the presence or absence of fenofibrate (50 μM) or 3-MA (3 mM). The amounts of LC3 I, and LC3 II (**A**), p62 (**B**), cleaved caspase-3 **(C)**, cleaved caspase-8 **(D)** and β-actin were measured by immunoblot analyses and normalized to the levels of the indicated proteins. **(A**–**D)** a representative blot is shown in the upper panel. Results are expressed as the mean ± SD of a representative experiment (n = 3 for each group). NS: not significant, and * P < 0.05, **P < 0.01, significantly different from cells incubated under the indicated conditions according to 1-way ANOVA with Scheffe's (**A-D**) post hoc comparison.

Considering the previously reported autophagy-promoting effects of fenofibrate [14], these effects were also evaluated in mProx cells. Compared with the cells under basal conditions, starvation for 3 or 6 h increased autophagy, reflected by an increase in LC3 II/I (Fig. 4A) and a decrease in p62 (Fig. 4B). Fenofibrate further altered the starvation-induced changes in LC3 II/I and p62 (Fig. 4A and B) despite increased p62 mRNA levels (Fig. S18). p-AMPK, an enhancer of autophagy initiation [14], was also upregulated by fenofibrate under basal and starvation conditions (Fig. S19).

### 3.4 Fenofibrate suppressed caspase activation with little involvement of autophagy in mProx cells

As the protective effects of autophagy on cisplatin nephropathy were previously reported [7,9], we examined whether autophagy inhibition increased cisplatin-induced apoptosis in the mProx cells treated with fenofibrate. An autophagy inhibitor, 3-MA (3 mM), increased cisplatin-induced caspase-3 and -8 activation by 2.1- and 2.2-fold, respectively (Fig. 4C and D). However, the fenofibrate-induced inhibition rates of caspase-3 and -8 activation compared with basal conditions not differ in cisplatin-treated mProx cells with and without 3-MA use (Fig. 4C and D). This suggested that fenofibrate attenuated cisplatin-induced caspase activation with little involvement of autophagy in the mProx cells.

## 4 Discussion

In the present study, we demonstrated that fenofibrate significantly reduces cisplatin-induced apoptosis of mProx cells by attenuating caspase-3 activation through the suppression of p53/Puma/Cytochrome C pathways and caspase-8 activation by suppressing MAPK, NFkB and death receptor apoptotic pathways. Although fenofibrate promoted autophagy by increasing p-AMPK, the inhibitory effects of caspase-3 and -8 activation were unaffected by autophagy inhibition by 3-MA. Fenofibrate also reduced the cisplatin-induced activation of PDK-4 that causes mitochondrial dysfunction [21]. This suggests that fenofibrate protects against cisplatin-induced renal tubular cell injury by inhibiting mitochondrial and death receptor apoptotic pathways with little involvement of autophagy.

In the mProx cells, fenofibrate significantly reduced cisplatin-stimulated caspase-9 activation specifically involved in the mitochondrial apoptotic pathway. Puma and Bax, p53-induced proapoptotic proteins, cause mitochondrial damage [1,23]. The two molecules translocate from the cytosol to mitochondria, causing mitochondrial membrane injury through Puma-promoted Bax activation [23], whereas Puma or Bax knockout ameliorates cisplatin-induced apoptosis in renal tubular cells [1,4]. Cytosolic protein 14-3-3 sequesters Bax in the cytosol [20]. JNK-mediated phosphorylation of 14-3-3 may promote its dissociation from Bax and subsequent Bax translocation to mitochondria [20]. Similarly, 14-3-3 also interacts with Bad [24]. As Bad and Puma belong to a BH3-only protein family, 14-3-3 may be also a cytosolic binder for Puma as it is for Bax and Bad. Considering the above, it is plausible that fenofibrate reduced mitochondrial apoptotic pathways by suppressing the phosphorylation of p53, JNK and 14-3-3, and consequently reducing Puma expression and mitochondrial Puma accumulation in the mProx cells. Bezafibrate, a fibrate class of PPAR-α ligand, was reported to prevent cisplatin-induced tubular apoptosis by reducing mitochondrial Bax accumulation and resultant mitochondrial damage [13].

In the present study, fenofibrate also attenuated cisplatin-stimulated caspase-8 activation in the mProx cells. Fenofibrate inhibited not only cisplatin-induced MAPK and NFkB signaling pathways, but also cisplatin-activated death receptor pathways. In earlier studies using LLC-PK1 and HK-2 cell lines, fenofibrate reduced cisplatin-induced apoptosis by inhibiting JNK and p38 pathways [12]. In mice, WY-14,643, another fibrate class, protected against cisplatin-induced nephropathy by reducing the expression of pro-inflammatory cytokines and chemokines [11]. In addition to supporting these previous findings, our study demonstrated that fenofibrate suppresses the death receptor pathways and downstream caspase-8 activation.

Autophagy is induced before caspase activation and apoptosis in cisplatin nephrotoxicity [6,7], and autophagy inhibition increases cisplatin-induced caspase activation and apoptosis [6,7,9,25]. These findings were also confirmed in the mProx cells in our study. Furthermore, appropriate augmentation of autophagy takes a protective role in the injury of renal proximal tubular cells *in vitro* and *in vivo* [8,25]. More recently, PPAR-α activation and its ligand, fibrates, were reported to alter autophagy [15]. In our study, fenofibrate promoted autophagy in mProx cells under starvation. However, autophagy inhibition had no influence on the inhibitory effects of fenofibrate on caspase-3 and -8 activation. Therefore, the inhibitory effects have little involvement in the promotion of autophagy in the mProx cells.

Additionally, there were some potential limitations of the adopted methodology in our study. In many experiments, only one type of approach, i.e., immunoblot analysis was used to quantify target proteins. Main conclusions were drawn from these data and were not verified by another approach. It would be preferable if we could have used two dimensional approach for protein quantification. In autophagy inhibition experiments, some reagents other than 3 MA, such as ATG5 or 7 siRNA should have been used to make the results more significant.

In conclusion, fenofibrate protected against cisplatin-induced injury in mProx cells by inhibiting the p53/Puma/Caspase-9/Caspase-3 pathway and the MAPK/Death receptor/Caspase-8 pathway, with little involvement of its autophagy-promoting effects. Therefore, fenofibrate may be a candidate agent to mitigate cisplatin nephrotoxicity.

## Declaration of competing interest

The authors declare that they have no known competing financial interests or personal relationships that could have appeared to influence the work in this paper.
