# Supplementary material for: Fenofibrate reduces cisplatin-induced apoptosis by inhibiting the p53/Puma/Caspase-9 pathway and the MAPK/Caspase-8 pathway rather than by promoting autophagy in murine renal proximal tubular cells
Source: Biochem Biophys Rep. 2022 Feb 28;30:101237. doi: 10.1016/j.bbrep.2022.101237 (PMC8889369; doi:10.1016/j.bbrep.2022.101237)
Supplement: Multimedia component 1 [file mmc1.docx]

**Supplementary Materials**

**Methods**

**Materials**

Rabbit monoclonal antibodies against mouse cleaved caspase-8, mouse p53, phosphorylated mouse p53 (p-p53; Ser15), human p53 upregulated modulator of apoptosis (Puma), human PDH, human phosphorylated PDH (p-PDH), and human phosphorylated AMPK (p-AMPK), and rabbit polyclonal antibodies against human cleaved caspase-3, mouse caspase-12 and human AMPK were purchased from Cell Signaling Technology (Boston, MA, USA). Rabbit polyclonal antibodies against human cellular FADD-like IL-1beta-converting enzyme inhibitory protein (c-FLIP) and β-actin and rabbit monoclonal antibodies against human cytochrome C were purchased from Abcam Inc. (Cambridge, UK).

**Tubular cell cultures**

mProx cells were generated as previously described [[1](#_ENREF_1)]. These cells were grown in modified K-1 medium (50:50 Ham’s F-12/DMEM) with 10% FBS, 5% CO_2_ and 95% air in a humidified atmosphere at 37.0°C. mProx cells (passage 10 through 14) were seeded on 12-well plates as appropriate. The modified K-1 medium was renewed every 2 days until semi-confluence was achieved. The medium was renewed immediately before the stimulation experiment. Fenofibrate and cisplatin were prepared as stock solutions in dimethyl sulfoxide (DMSO) and 0.9% NaCl, respectively, and further diluted to working concentrations in the cell incubation medium. The final concentration of DMSO in our experiments did not exceed 0.1%.

**TaqMan real-time PCR assay**

The TaqMan real-time PCR assay was performed using a StepOnePlus Real-Time PCR System (Thermo Fisher Scientific Inc., Waltham, MA USA) as previously reported [[2](#_ENREF_2)]. Unlabeled specific primers and TaqMan MGB probes (6-FAM dye-labeled) from Applied Biosystems were used to detect mouse Bax (Mm00432051_m1), Puma (Mm00519268_m1), PDK4 (Mm01166879_m1), Catalase (Mm00437992_m1), SOD2 (Mm01313000_m1), PPAR-α (Mm 00440939_m1), TL1A (Mm00770031_m1), FAS (Mm04206620_m1) and p62 (Mm00448091_m1). The mRNA levels of each gene were normalized to β2-microglobulin mRNA levels.

**Immunoblot analysis**

Immunoblot analyses were performed as previously described [[2](#_ENREF_2)]. Whole cell lysates of mProx cells were prepared using RIPA buffer containing phosphatase inhibitors (Santa Cruz Biotechnology, CA, USA) according to the manufacturer’s instructions. The membranes to which proteins from mProx were transferred were incubated with anti-cleaved caspase-3 and -8, and caspase-12 (1:1000 dilution), anti-p53 (1:1000), anti-p-p53 (1:1000), anti-Puma (1:1000), anti-cytochrome C (1:5000), anti-PDH (1:1000 dilution), anti-p-PDH (1:2000 dilution), c-FLIP (1:1000 dilution), anti-AMPK (1:1000), anti-p-AMPK (1:1000), and anti-β actin (1:6000 dilution) antibodies for 20 min at room temperature. These membranes were then incubated with appropriate horseradish peroxidase-conjugated secondary antibodies (1:1000 dilution) at room temperature for 1 hour. Secondary antibodies were detected using ECL reagents.


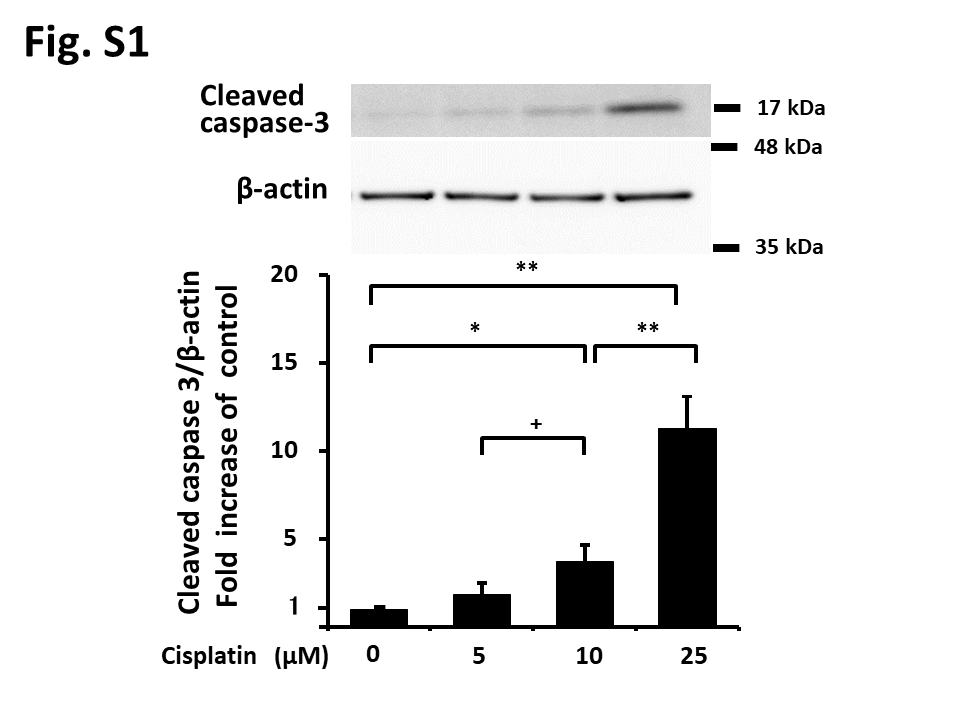


**Fig. S1**

Figure S1

Cisplatin increased cleaved caspase-3 amounts dose-dependently in mProx cells. mProx cells were incubated for 24 hours in modified K-1 medium with cisplatin (0, 5, 10 and 25 μM). The amounts of cleaved caspase-3 were measured by immunoblot analysis and normalized to the β-actin levels. The average cleaved caspase-3 amount in mProx cells untreated was set to 1.0. A representative blot is shown in the upper panel. Results are expressed as the mean ± SD of a representative experiment (n=3 for each group). + P<0.08, * P<0.05, ** P<0.01, significantly different from cells incubated under the indicated conditions, according to analysis of variance with Scheffe's post hoc comparison.

**Fig. S2**


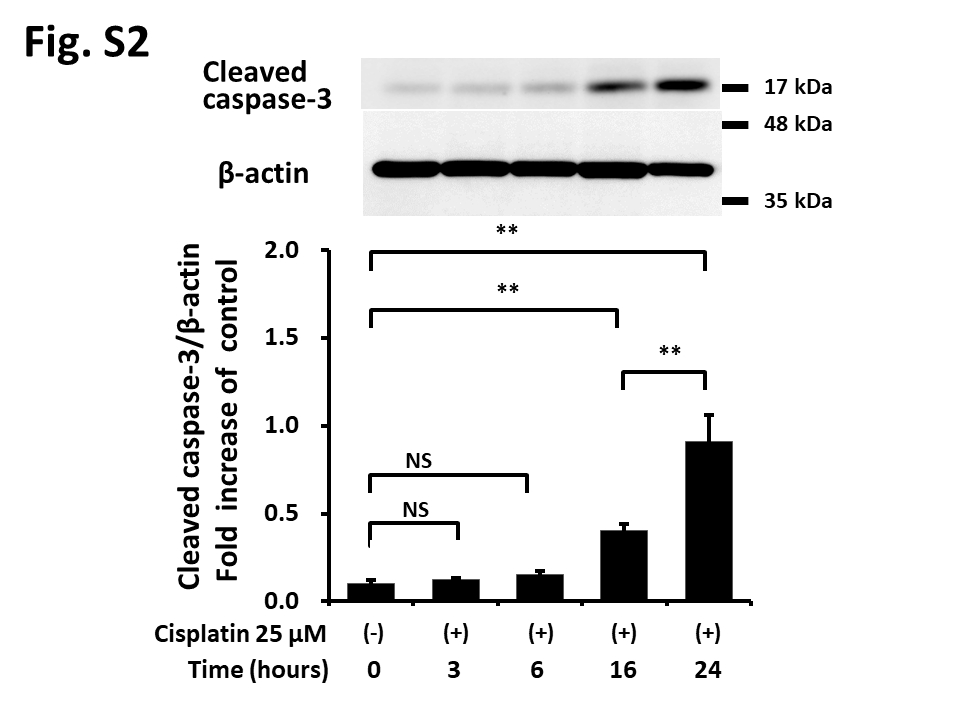


Figure S2

Cisplatin increased cleaved caspase-3 amounts time-dependently in mProx cells. mProx cells were incubated for 0, 3, 6, 16 and 24 hours in modified K-1 medium with or without cisplatin (25 μM). The amounts of cleaved caspase-3 were measured by immunoblot analysis and normalized to the β-actin levels. The average cleaved caspase-3 amount in mProx cells untreated was set to 1.0. A representative blot is shown in the upper panel. Results are expressed as the mean ± SD of a representative experiment (n=3 for each group). NS: not significant, and ** P<0.01, significantly different from cells incubated under the indicated conditions, according to analysis of variance with Scheffe's post hoc comparison.


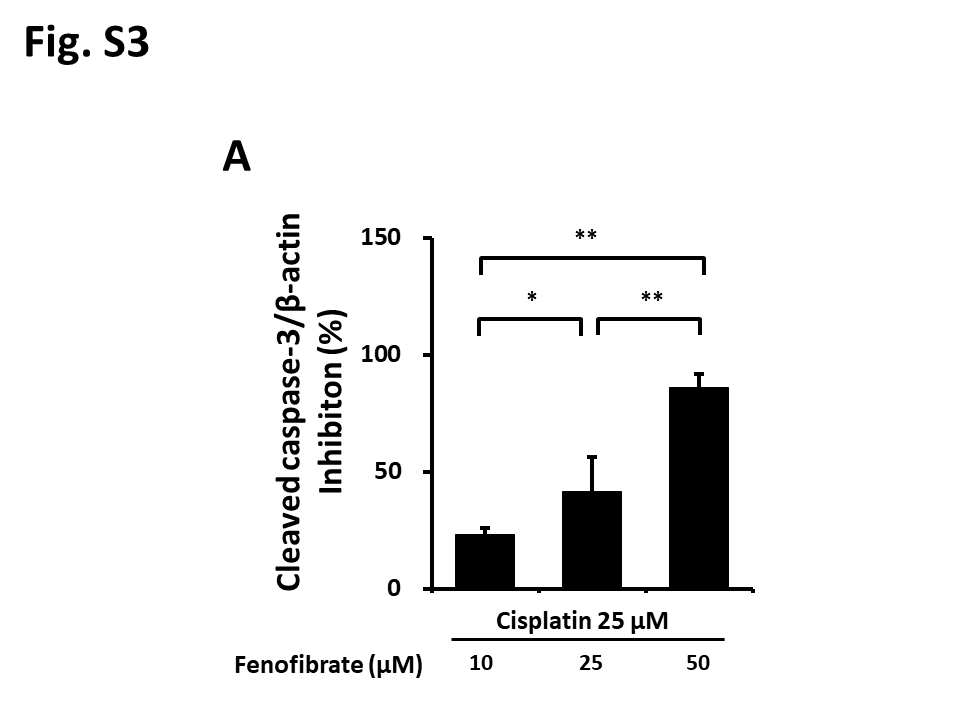


**Fig. S3**


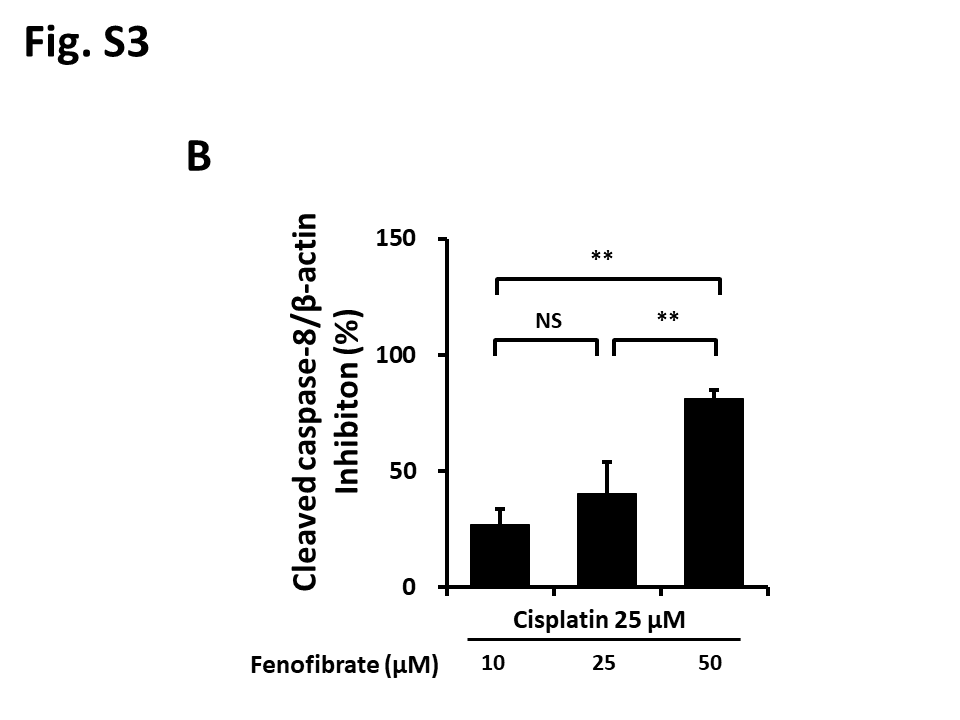


**Fig. S3**

Figure S3

Fenofibrate increased the percentage reduction of cisplatin-induced caspase-3 and -8 activation dose-dependently in mProx cells. mProx cells were incubated for 24 hours in modified K-1 medium with or without cisplatin (25 μM) in the presence or absence of fenofibrate (0, 10, 25 and 50 μM). The amounts of cleaved caspase-3 (A) and -8 (B) were measured by immunoblot analysis and normalized to the β-actin levels. The percentage reduction of cleaved caspases (% reduction) was calculated by the following formula: % reduction = (a-b)×100/a, where a and b are cisplatin-induced increments in cleaved caspase-3 or -8 amounts in mProx cells without and with fenofibrate, respectively. Results are expressed as the mean ± SD of 3 independent experiments. NS: not significant, and * P<0.05, ** P<0.01, significantly different from cells incubated under the indicated conditions, according to analysis of variance with Fisher's post hoc comparison.

Values of inhibition (%) shown in Figs. S3A and S3B were calculated from uncropped immunoblot images for Figs. S3A and S3B shown below.


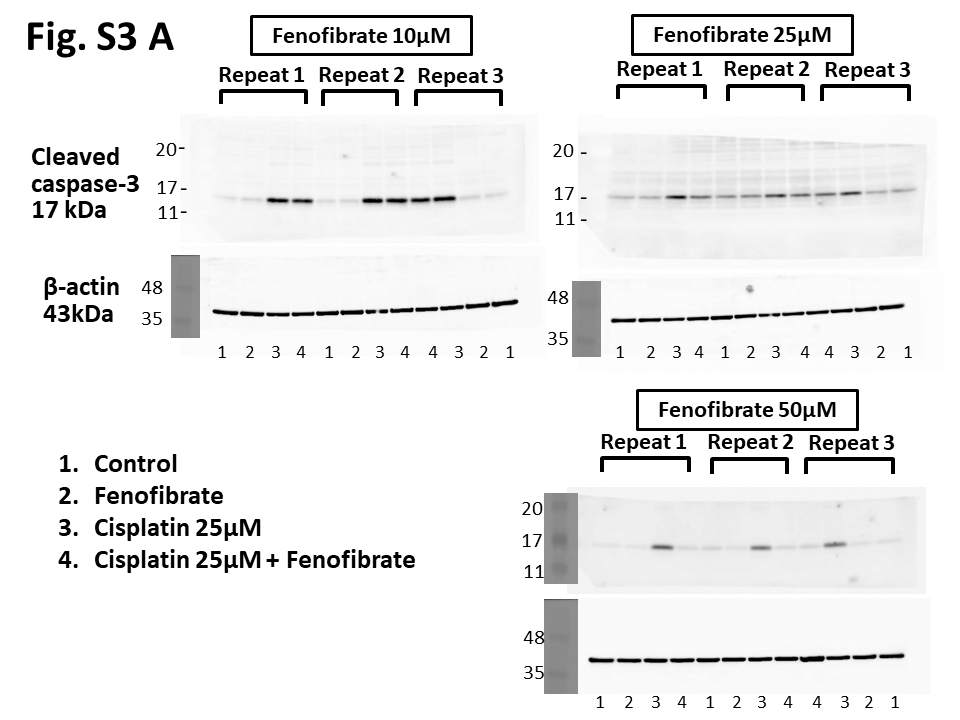


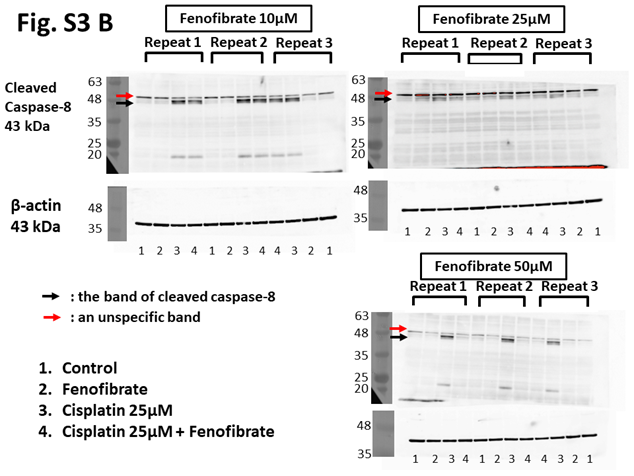


**Fig. S4**


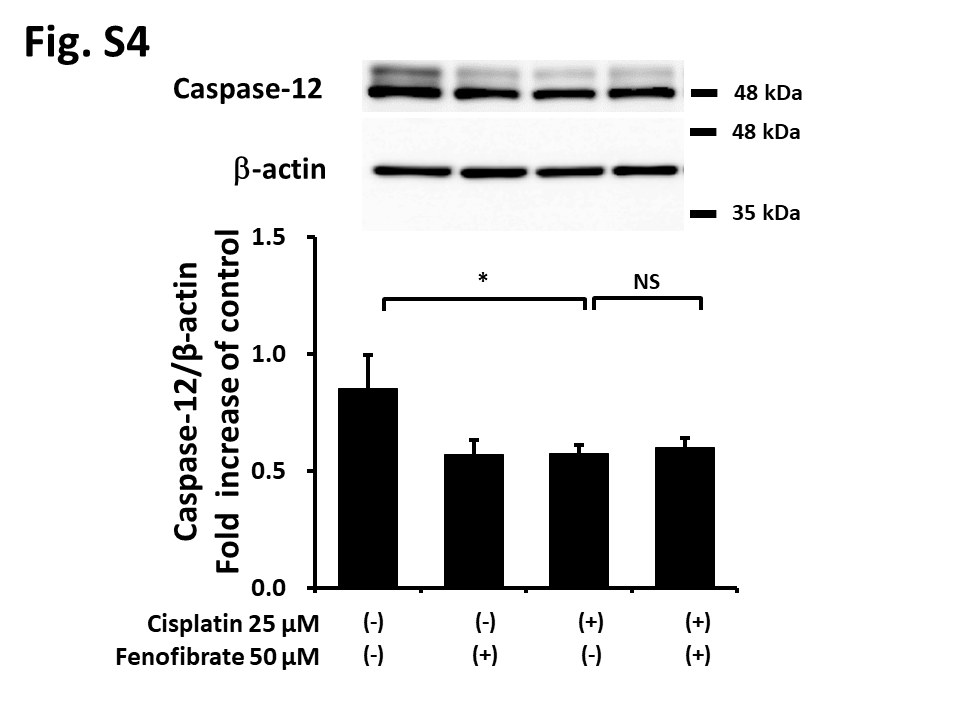


Figure S4

Fenofibrate did not reduce cisplatin-induced caspase-12 activation in mProx cells. mProx cells were incubated for 24 hours in modified K-1 medium with or without cisplatin (25 μM) in the presence or absence of fenofibrate (50 μM). The amounts of caspase-12 were measured by immunoblot analysis and normalized to the β-actin levels. The reduction of caspase-12 was considered caspase-12 activation. A representative blot is shown in the upper panel. Results are expressed as the mean ± SD of a representative experiment (n=3 for each group). NS: not significant, and * P<0.05, significantly different from cells incubated under the indicated conditions, according to analysis of variance with Scheffe's post hoc comparison.


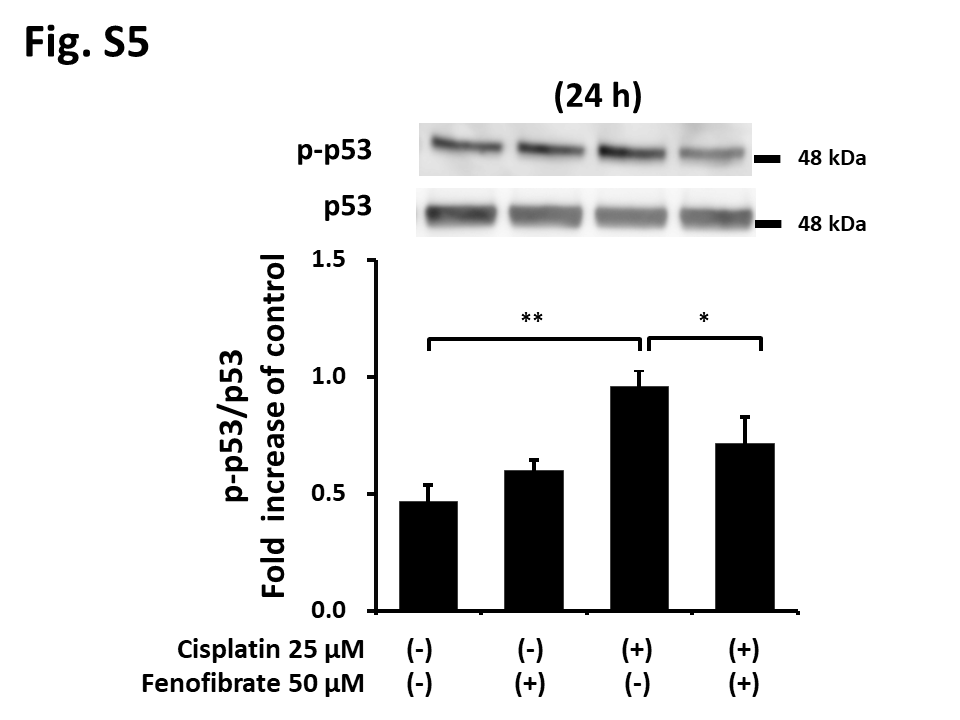


**Fig. S5**

Figure S5

Fenofibrate reduced cisplatin-induced phosphorylation of p53 in mProx cells. mProx cells were incubated for 24 hours in modified K-1 medium with or without cisplatin (25 μM) in the presence or absence of fenofibrate (50 μM). The amounts of phosphorylated p53 (p-p53) were measured by immunoblot analyses and normalized to the p53 levels. A representative blot is shown in the upper panel. Results are expressed as the mean ± SD of a representative experiment (n=3 for each group). * P<0.05, ** P<0.01, significantly different from cells incubated under the indicated conditions, according to analysis of variance with Scheffe's post hoc comparison.


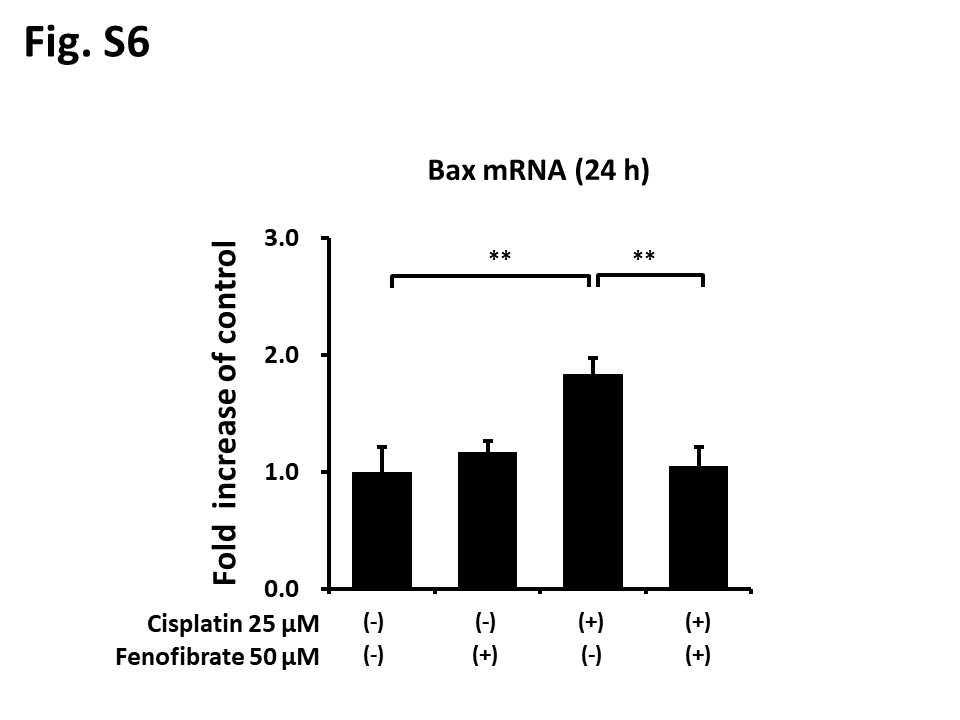


**Fig. S6**

Figure S6

Fenofibrate reduced cisplatin-increased Bax mRNA levels in mProx cells. mProx cells were incubated for 24 hours in modified K-1 medium with or without cisplatin (25 μM) in the presence or absence of fenofibrate (50 μM). The levels of Bax mRNA were measured by real-time PCR assay and normalized to the levels of β2-microglobulin mRNA. The average Bax mRNA level in untreated cells was set to 1.0. Results are expressed as the mean ± SD of a representative experiment (n=3 for each group).

** P<0.01, significantly different from cells incubated under the indicated conditions, according to analysis of variance with Scheffe's post hoc comparison.


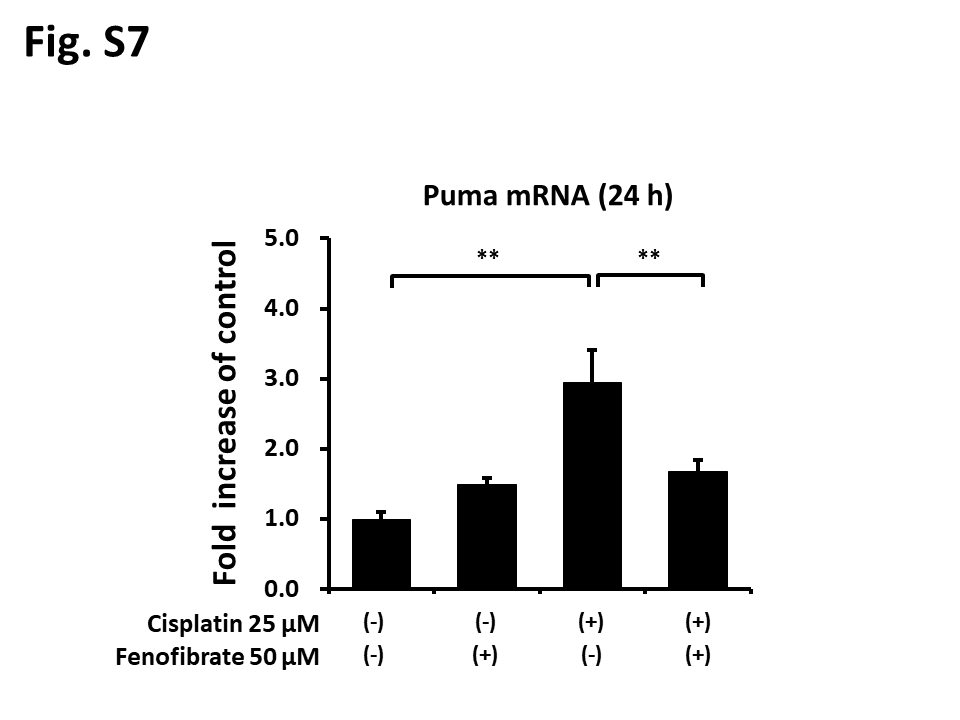


**Fig. S7**

Figure S7

Fenofibrate reduced cisplatin-increased Puma mRNA levels in mProx cells. mProx cells were incubated for 24 hours in modified K-1 medium with or without cisplatin (25 μM) in the presence or absence of fenofibrate (50 μM). The levels of Puma mRNA were measured by real-time-PCR assay and normalized to the levels of β2-microglobulin mRNA. The average Puma mRNA level in untreated cells was set to 1.0. Results are expressed as the mean ± SD of a representative experiment (n=3 for each group).

** P<0.01, significantly different from cells incubated under the indicated conditions, according to analysis of variance with Scheffe's post hoc comparison.


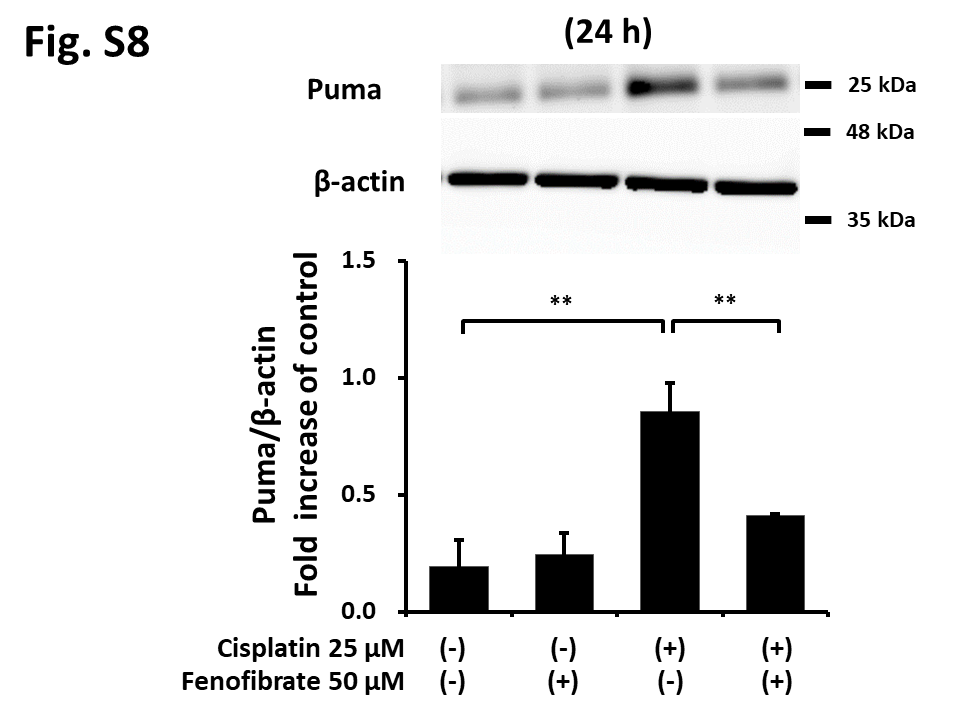


Figure S8

Fenofibrate reduced cisplatin-increased amounts of cellular Puma in mProx cells. mProx cells were incubated for 24 hours in modified K-1 medium with or without cisplatin (25 μM) in the presence or absence of fenofibrate (50 μM). The amounts of Puma were measured by immunoblot analyses and normalized to the β-actin levels. A representative blot is shown in the upper panel. Results are expressed as the mean ± SD of a representative experiment (n=3 for each group). ** P<0.01, significantly different from cells incubated under the indicated conditions, according to analysis of variance with Scheffe's post hoc comparison.


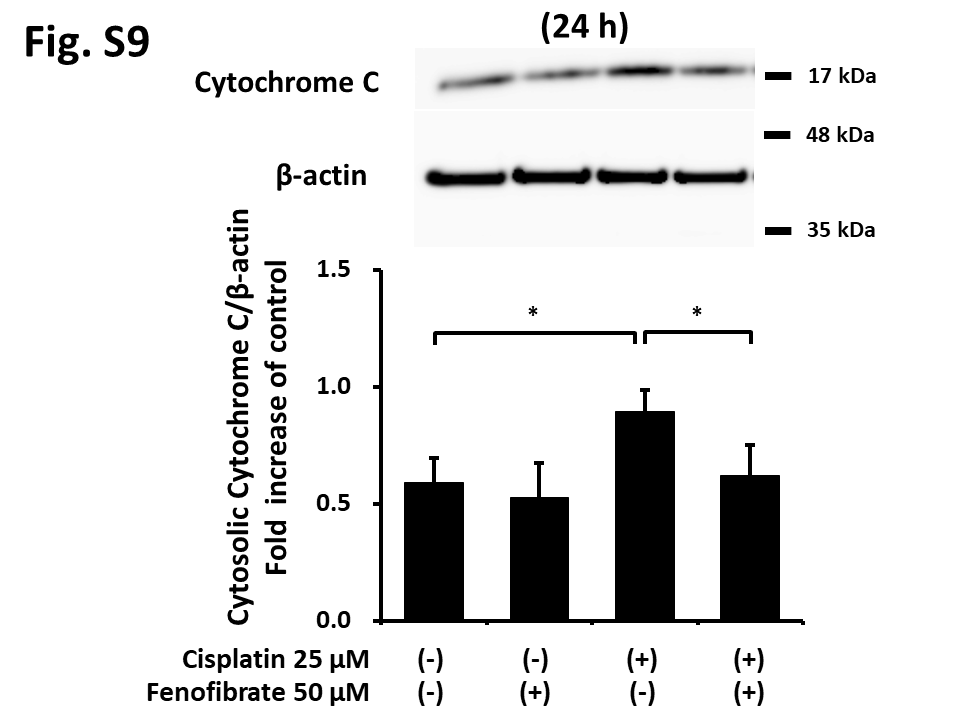


Figure S9

Fenofibrate reduced cisplatin-increased amounts of cytosolic cytochrome C in mProx cells. mProx cells were incubated for 24 hours in modified K-1 medium with or without cisplatin (25 μM) in the presence or absence of fenofibrate (50 μM). The amounts of cytosolic cytochrome C were measured by immunoblot analyses and normalized to the β-actin levels. A representative blot is shown in the upper panel. Results are expressed as the mean ± SD of a representative experiment (n=3 for each group). * P<0.05, significantly different from cells incubated under the indicated conditions, according to analysis of variance with Fisher’s post hoc comparison.


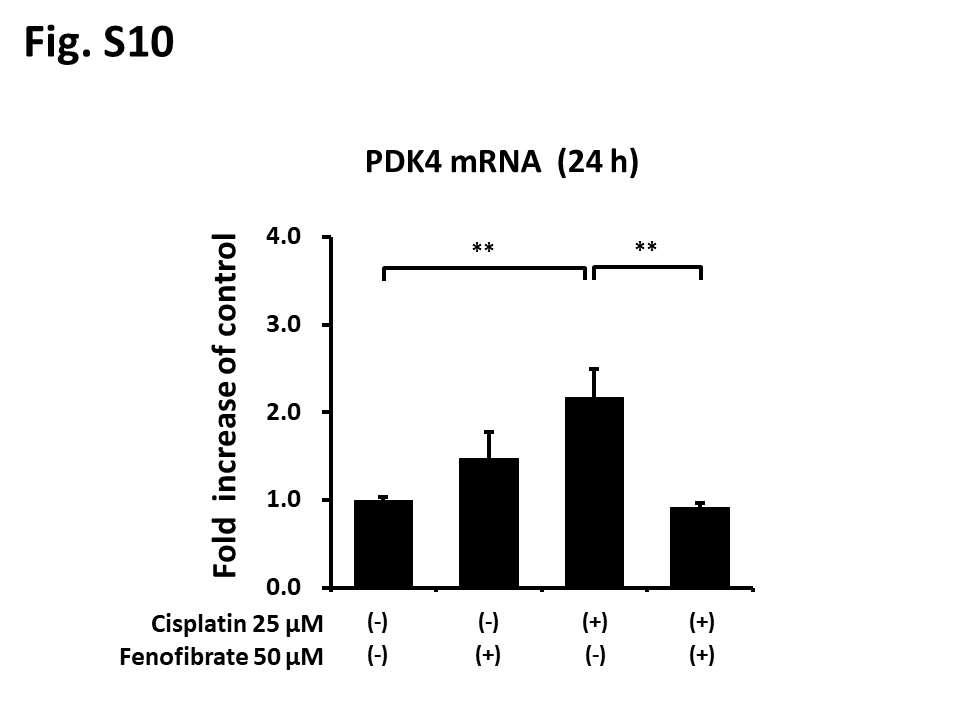


**Fig. S10**

Figure S10

Fenofibrate reduced cisplatin-increased PDK4 mRNA levels in mProx cells. mProx cells were incubated for 24 hours in modified K-1 medium with or without cisplatin (25 μM) in the presence or absence of fenofibrate (50 μM). The levels of PDK4 mRNA were measured by real-time PCR assay and normalized to the levels of β2-microglobulin mRNA. The average PDK4 mRNA level in untreated cells was set to 1.0. Results are expressed as the mean ± SD of a representative experiment (n=3 for each group).

** P<0.01, significantly different from cells incubated under the indicated conditions, according to analysis of variance with Scheffe's post hoc comparison.


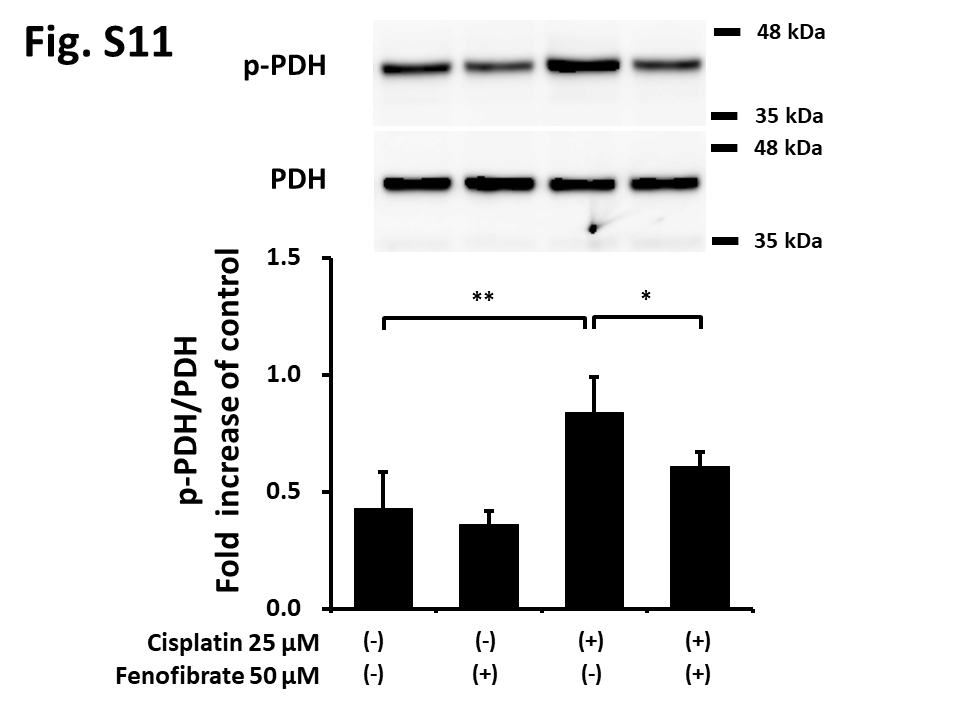


**Fig. S11**

Figure S11

Fenofibrate reduced cisplatin-increased p-PDH/PDH values in mProx cells. mProx cells were incubated for 24 hours in modified K-1 medium with or without cisplatin (25 μM) in the presence or absence of fenofibrate (50 μM). The amounts of p-PDH and PDH were measured by immunoblot analysis. A representative blot is shown in the upper panel. Results are expressed as the mean ± SD of a representative experiment (n=3 for each group). * P<0.05, ** P<0.01, significantly different from cells incubated under the indicated conditions, according to analysis of variance with Fisher's post hoc comparison.


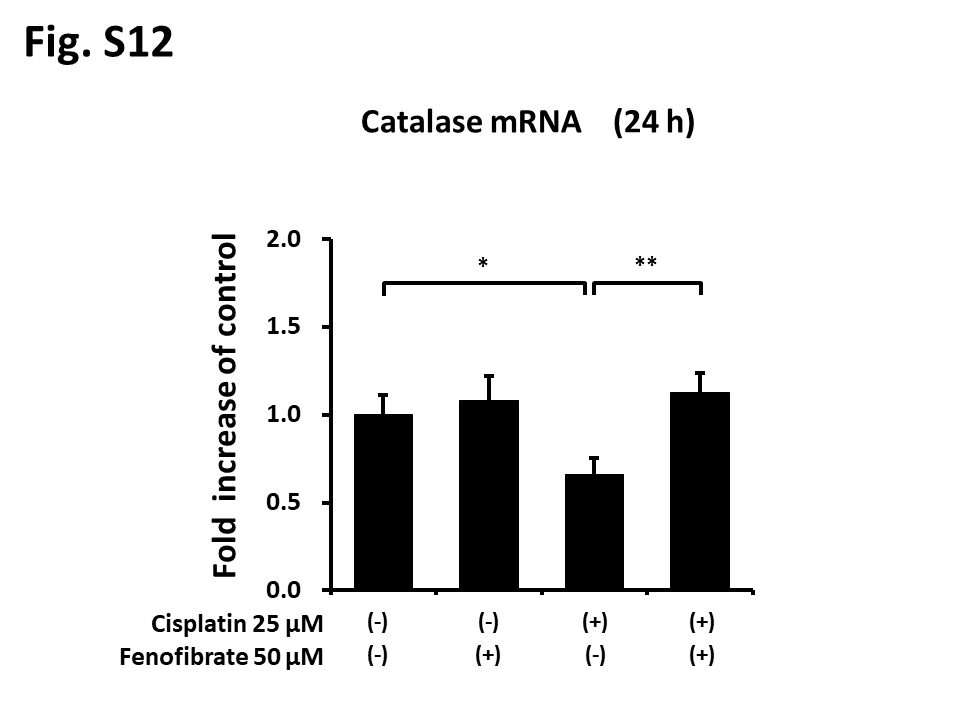


**Fig. S12**

Figure S12

Fenofibrate increased cisplatin-reduced levels of catalase mRNA in mProx cells. mProx cells were incubated for 24 hours in modified K-1 medium with or without cisplatin (25 μM) in the presence or absence of fenofibrate (50 μM). The levels of catalase mRNA were measured by real-time PCR assay and normalized to the levels of β2-microglobulin mRNA. The average catalase mRNA level in untreated cells was set to 1.0. Results are expressed as the mean ± SD of a representative experiment (n=3 for each group).

* P<0.05, ** P<0.01, significantly different from cells incubated under the indicated conditions, according to analysis of variance with Scheffe's post hoc comparison.


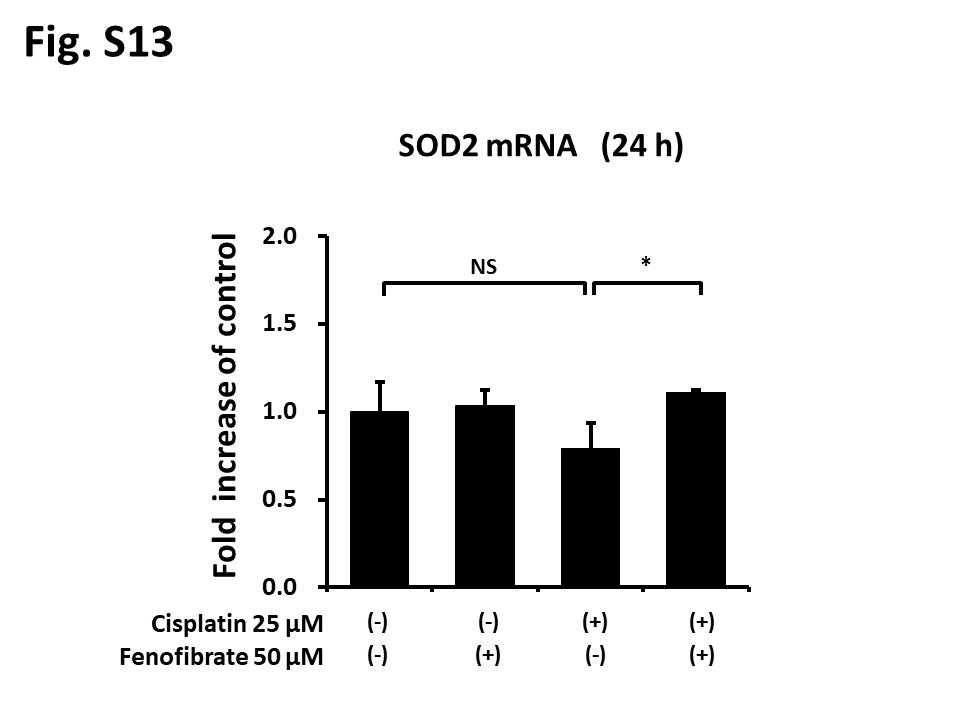


**Fig. S13**

**Fig. S10**

Figure S13

Fenofibrate increased cisplatin-reduced levels of SOD2 mRNA in mProx cells. mProx cells were incubated for 24 hours in modified K-1 medium with or without cisplatin (25 μM) in the presence or absence of fenofibrate (50 μM). The levels of SOD2 mRNA were measured by real-time PCR assay and normalized to the levels of β2-microglobulin mRNA. The average catalase mRNA level in untreated cells was set to 1.0. Results are expressed as the mean ± SD of a representative experiment (n=3 for each group). NS: not significant, and * P<0.05, significantly different from cells incubated under the indicated conditions, according to analysis of variance with Fisher's post hoc comparison.


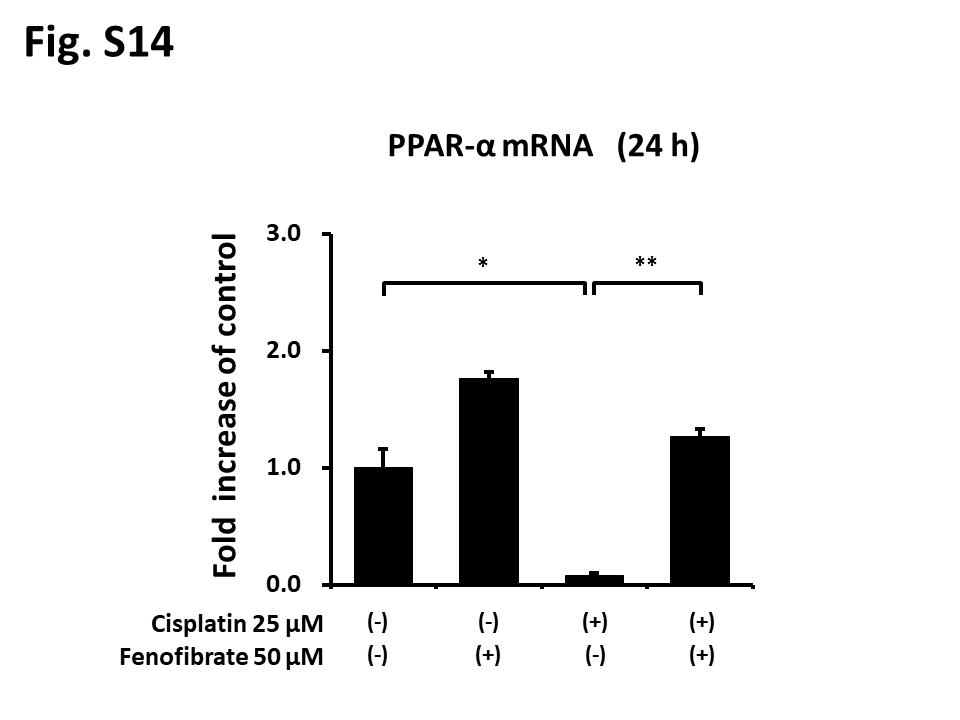


**Fig. S14**

Figure S14

Fenofibrate increased cisplatin-reduced levels of PPAR-α mRNA in mProx cells. mProx cells were incubated for 24 hours in modified K-1 medium with or without cisplatin (25 μM) in the presence or absence of fenofibrate (50 μM). The levels of PPAR-α mRNA were measured by TaqMan PCR assay and normalized to the levels of β2-microglobulin mRNA. The average PPAR-α mRNA level in mProx cells under basal conditions was set to 1.0. Results are expressed as the mean ± SD of a representative experiment (n=3 for each group). * P<0.05, ** P<0.01, significantly different from cells incubated under the indicated conditions, according to analysis of variance with Scheffe's post hoc comparison.


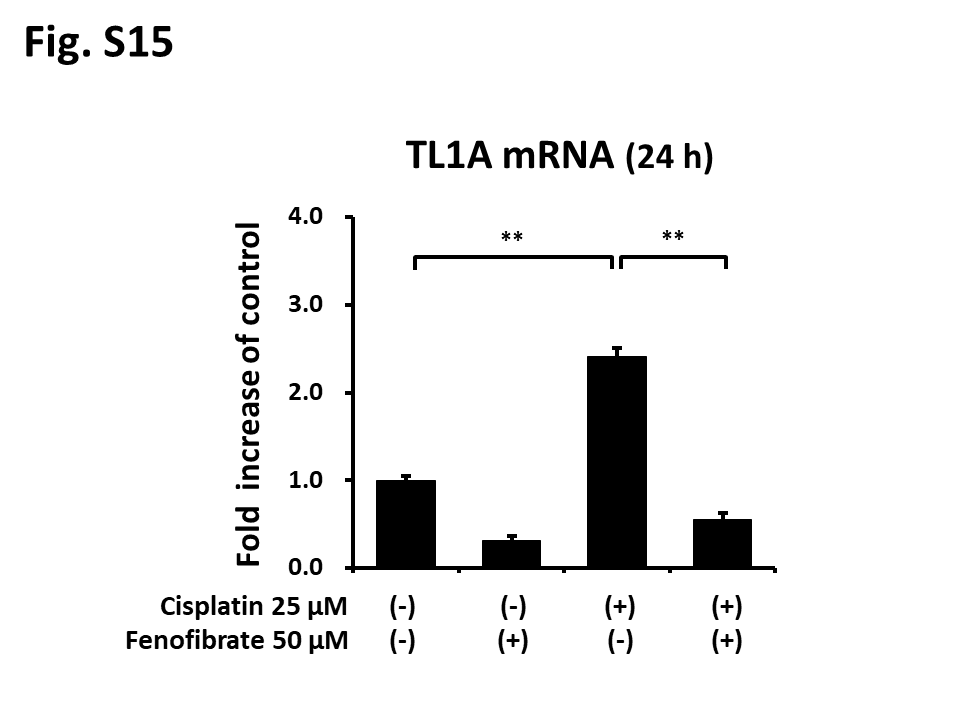


Figure S15

Fenofibrate increased cisplatin-reduced levels of TL1A mRNA in mProx cells. mProx cells were incubated for 24 hours in modified K-1 medium with or without cisplatin (25 μM) in the presence or absence of fenofibrate (50 μM). The levels of TL1A mRNA were measured by TaqMan PCR assay and normalized to the levels of β2-microglobulin mRNA. The average TL1A mRNA level in mProx cells under basal conditions was set to 1.0. Results are expressed as the mean ± SD of a representative experiment (n=3 for each group). ** P<0.01, significantly different from cells incubated under the indicated conditions, according to analysis of variance with Scheffe's post hoc comparison.


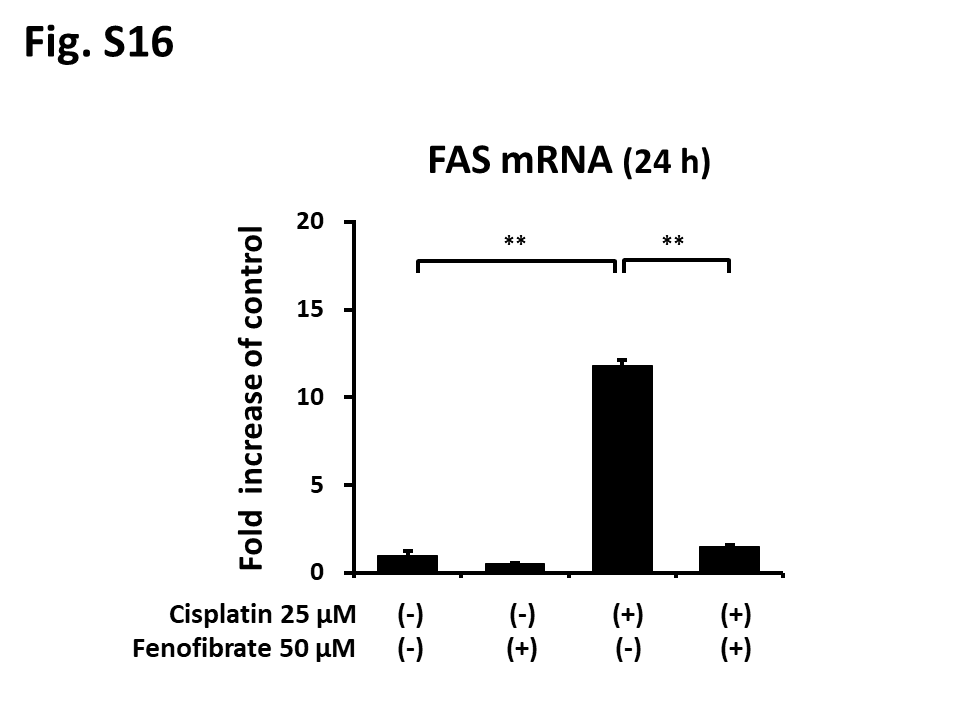


Figure S16

Fenofibrate increased cisplatin-reduced levels of Fas mRNA in mProx cells. mProx cells were incubated for 24 hours in modified K-1 medium with or without cisplatin (25 μM) in the presence or absence of fenofibrate (50 μM). The levels of Fas mRNA were measured by TaqMan PCR assay and normalized to the levels of β2-microglobulin mRNA. The average Fas mRNA level in mProx cells under basal conditions was set to 1.0. Results are expressed as the mean ± SD of a representative experiment (n=3 for each group). ** P<0.01, significantly different from cells incubated under the indicated conditions, according to analysis of variance with Scheffe's post hoc comparison.


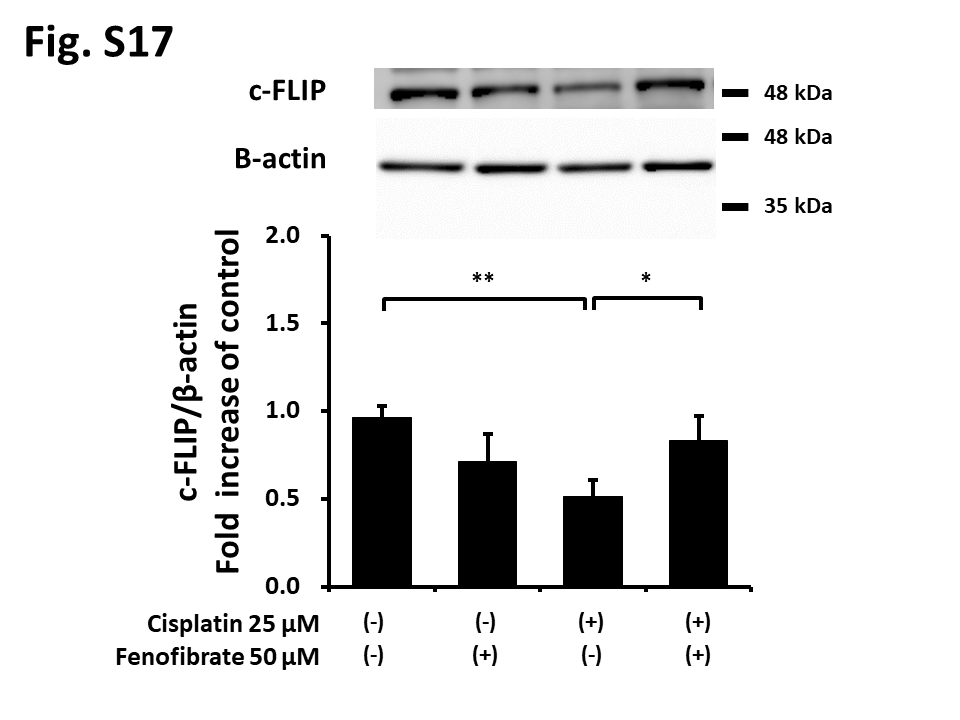


**Fig. S17**

Figure S17

Fenofibrate increased cisplatin-reduced c-FLIP amounts in mProx cells. mProx cells were incubated for 24 hours in modified K-1 medium with or without cisplatin (25 μM) in the presence or absence of fenofibrate (50 μM). The amounts of c-FLIP were measured by immunoblot analysis and normalized to β-actin levels. Results are expressed as the mean ± SD of a representative experiment (n=3 for each group). * P<0.05, ** P<0.01, significantly different from cells incubated under the indicated conditions, according to analysis of variance with Fisher's post hoc comparison.


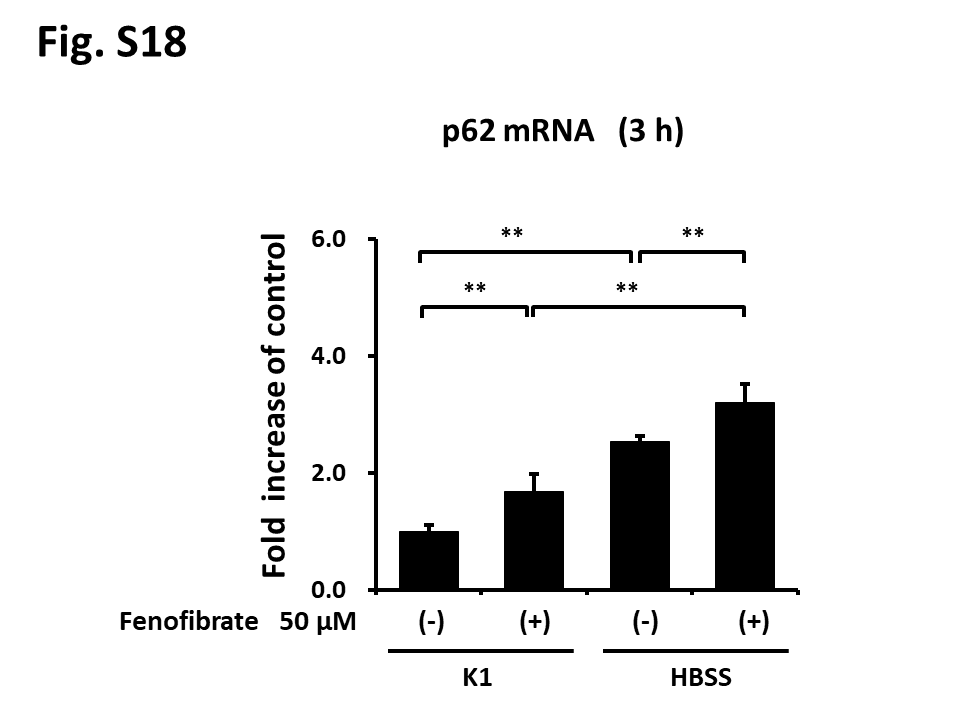


Figure S18

Fenofibrate increased p62 mRNA levels in mProx cells under basal and fasting conditions. Confluent mProx cells were incubated for 3 hours in modified K-1 medium or HBSS medium in the presence or absence of fenofibrate (50 μM). The level of p62 mRNA was measured by real-time PCR assay and normalized to β2-microglobulin mRNA levels. The average PPAR-α mRNA level in mProx cells under basal conditions was set to 1.0. Results are expressed as the mean ± SD of a representative experiment (n=3 for each group). ** P<0.01, significantly different from cells incubated under the indicated conditions, according to analysis of variance with Scheffe's post hoc comparison.


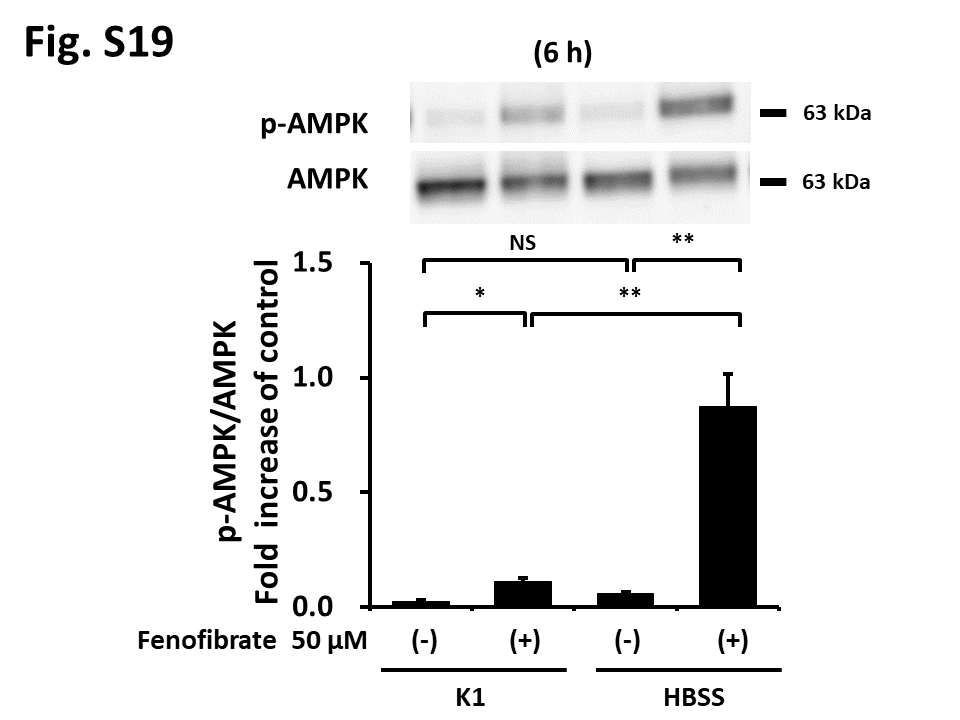


Figure S19

Fenofibrate increased cisplatin-increased values of p-AMPK/AMPK in mProx cells under basal and fasting conditions. Confluent mProx cells were incubated for 3 hours in modified K-1 medium or HBSS medium in the presence or absence of fenofibrate (50 μM). The amounts of p-AMPK were measured by immunoblot analysis and normalized to the AMPK levels. A representative blot is shown in the upper panel. Results are expressed as the mean ± SD of a representative experiment (n=3 for each group). NS: not significant, ** P<0.01, significantly different from cells incubated under the indicated conditions, according to analysis of variance with Scheffe's post hoc comparison.

* P<0.05, significantly different from cells incubated under the indicated conditions according to the Student’s t-test.

References

[1] H. Okada, T. Kikuta, T. Inoue, Y. Kanno, S. Ban, T. Sugaya, M. Takigawa, H. Suzuki, Dexamethasone induces connective tissue growth factor expression in renal tubular epithelial cells in a mouse strain-specific manner, Am J Pathol 168 (2006) 737-747.

[2] D. Mikami, H. Kimura, K. Kamiyama, K. Torii, K. Kasuno, N. Takahashi, H. Yoshida, M. Iwano, Telmisartan activates endogenous peroxisome proliferator-activated receptor-delta and may have anti-fibrotic effects in human mesangial cells, Hypertens Res 37 (2014) 422-431.
